# Identifying brain targets for real-time fMRI neurofeedback in chronic pain: insights from functional neurosurgery

**DOI:** 10.1093/psyrad/kkae026

**Published:** 2024-11-21

**Authors:** Dan Liu, Yiqi Mi, Menghan Li, Anna Nigri, Marina Grisoli, Keith M Kendrick, Benjamin Becker, Stefania Ferraro

**Affiliations:** Sichuan Provincial Center for Mental Health, Sichuan Provincial People's Hospital, University of Electronic Science and Technology of China, Chengdu 611731, China; Ministry of Education Key Laboratory for Neuroinformation, School of Life Science and Technology, University of Electronic Science and Technology, Chengdu 610054, China; Ministry of Education Key Laboratory for Neuroinformation, School of Life Science and Technology, University of Electronic Science and Technology, Chengdu 610054, China; Sichuan Provincial Center for Mental Health, Sichuan Provincial People's Hospital, University of Electronic Science and Technology of China, Chengdu 611731, China; Ministry of Education Key Laboratory for Neuroinformation, School of Life Science and Technology, University of Electronic Science and Technology, Chengdu 610054, China; Neuroradiology Department, Neurological Institute Carlo Besta, 20133 Milan, Italy; Neuroradiology Department, Neurological Institute Carlo Besta, 20133 Milan, Italy; Sichuan Provincial Center for Mental Health, Sichuan Provincial People's Hospital, University of Electronic Science and Technology of China, Chengdu 611731, China; Ministry of Education Key Laboratory for Neuroinformation, School of Life Science and Technology, University of Electronic Science and Technology, Chengdu 610054, China; State Key Laboratory of Brain and Cognitive Sciences, The University of Hong Kong, 999077 Hong Kong, China; Department of Psychology, The University of Hong Kong, 999077 Hong Kong, China; Sichuan Provincial Center for Mental Health, Sichuan Provincial People's Hospital, University of Electronic Science and Technology of China, Chengdu 611731, China; Ministry of Education Key Laboratory for Neuroinformation, School of Life Science and Technology, University of Electronic Science and Technology, Chengdu 610054, China

**Keywords:** rt-fMRI-NF, brain stimulation, sensorimotor network, salience network

## Abstract

**Background:**

The lack of clearly defined neuromodulation targets has contributed to the inconsistent results of real-time fMRI-based neurofeedback (rt-fMRI-NF) for the treatment of chronic pain. Functional neurosurgery (funcSurg) approaches have shown more consistent effects in reducing pain in patients with severe chronic pain.

**Objective:**

This study aims to redefine rt-fMRI-NF targets for chronic pain management informed by funcSurg studies.

**Methods:**

Based on independent systematic reviews, we identified the neuromodulation targets of the rt-fMRI-NF (in acute and chronic pain) and funcSurg (in chronic pain) studies. We then characterized the underlying functional networks using a subsample of the 7 T resting-state fMRI dataset from the Human Connectome Project. Principal component analyses (PCA) were used to identify dominant patterns (accounting for a cumulative explained variance >80%) within the obtained functional maps, and the overlap between these PCA maps and canonical intrinsic brain networks (default, salience, and sensorimotor) was calculated using a null map approach.

**Results:**

The anatomical targets used in rt-fMRI-NF and funcSurg approaches are largely distinct, with the middle cingulate cortex as a common target. Within the investigated canonical rs-fMRI networks, these approaches exhibit both divergent and overlapping functional connectivity patterns. Specifically, rt-fMRI-NF approaches primarily target the default mode network (*P* value range 0.001–0.002) and the salience network (*P* = 0.002), whereas funcSurg approaches predominantly target the salience network (*P* = 0.001) and the sensorimotor network (*P* value range 0.001–0.023).

**Conclusion:**

Key hubs of the salience and sensorimotor networks may represent promising targets for the therapeutic application of rt-fMRI-NF in chronic pain.

## Background

Functional neurosurgery (funcSurg), which includes techniques such as deep brain stimulation (DBS) and ablation of specific brain areas with different approaches (with surgical treatment, but also with the newer gamma-knife and MRgFUS or magnetic resonance-guided focused ultrasound), has a long tradition in treating severe chronic pain. Remarkable studies have provided evidence that stimulation or lesion of selected targets such as specific nuclei of the thalamus [notably the mediodorsal, and ventral posterior lateral and medial (VPL/VPM) and central lateral (CL) nuclei], the midbrain nuclei [periaqueductal gray (PAG)/periventricular gray (PVG) matter] and regions of the middle cingulate cortex (MCC) and primary motor cortex (Nguyen *et al*., 2011) can reduce pain levels and alleviate the emotional suffering associated with pain.

Over the past two decades, several meta-analyses on the use of DBS have offered different perspectives on the success of this approach in the treatment of chronic pain conditions. A recent comprehensive meta-analysis (Frizon *et al*., 2020) found that DBS has been predominantly used for central and peripheral neuropathic pain, with target areas identified in the PAG/PVG, VPL/VPM, and centromedian and parafascicular (CM/Pf) nuclei and, more recently, in cingulate regions (i.e. MCC). In their meta-analysis, (Bittar *et al*., 2005) demonstrated a reduction in long-term pain in a significant number of patients (79%) when targeting PAG/PVG areas; this percentage increases to 87% when both PAG/PVG and sensory thalamus (VPL/VPM) are involved. Regarding the success of DBS with respect to etiology, Bittar *et al*. (2005) showed greater efficacy of DBS in nociceptive pain (long-term pain control in 63% of patients but in 80% in patients with intractable low back pain) than in neuropathic pain (pain control in 47% of patients). Importantly, in the context of neuropathic pain, Frizon *et al*. (2020) showed that DBS for post-stroke (or central neuropathic) pain has a great variability in outcomes, with some authors reporting pain reduction in a substantial percentage of patients, while others highlighting that many patients did not receive complete implants or that DBS was not used in the long term. By contrast, DBS for peripheral neuropathic pain (from phantom limb or brachial plexus injury) induced significant pain improvement in a substantial percentage of patients (60–100%) across all the studies. Viswanathan *et al*. (2013), in their meta-analysis on anterior cingulotomy for the treatment of cancer pain, found that between 32 and 83% of patients experienced pain improvement, while a subsequent study (Boccard *et al*., 2017) showed that DBS in MCC could reduce neuropathic pain in all patients examined.

However, despite the remarkable results, the invasiveness of these brain-based interventions has inherently prevented their translation into routine clinical practice in the treatment of chronic pain, limiting their use to very severe cases.

In recent years, neurofeedback as a non-invasive form of brain modulation with a highly promising therapeutic potential has gained increasing interest. The recent development of real-time functional magnetic resonance imaging feedback (rt-fMRI-NF), leveraging on the relatively high spatial resolution of fMRI, can provide feedback on the activity of precise brain regions or networks (Grech *et al*., 2008). Training-induced changes in regional activity and pathways have been shown to induce specific changes in cognitive and emotional processes associated with these neural systems (Yao *et al*., 2016; Zhao *et al*., 2019), including acute pain (Emmert *et al*., 2014; Rance *et al*., 2014a,b). Notably, rt-fMRI-NF has shown promising results in the treatment of several psychiatric and neurological disorders (Chiba *et al*., 2019; Dudek and Dodell-Feder, 2021; Hamilton *et al*., 2016; Hanlon *et al*., 2013; Rance *et al*., 2023; Scheinost *et al*., 2013; Subramanian *et al*., 2016). These findings provide a solid theoretical and technical foundation for the development of rt-fMRI-NF in treating chronic pain conditions. So far, only two studies have investigated the effects of rt-fMRI-NF in chronic pain patients (DeCharms *et al*., 2005; Guan *et al*., 2015), and a few more have examined its impact on induced pain in healthy participants (Emmert *et al*., 2014; Rance *et al*., 2014a,b). While all these studies have provided evidence for the ability of individuals to learn to modulate the activity of specific brain regions with this technique, the failure to replicate previous results (DeCharms *et al*., 2005) and the lack of clear behavioral effects in acute pain (DeCharms *et al*., 2005; Emmert *et al*., 2014; Rance *et al*., 2014a,b), have induced a skepticism toward the potential role of rt-fMRI-NF in chronic pain management, thus limiting studies in this direction. However, the few findings suggest that it may be premature to dismiss rt-fMRI-NF in chronic pain treatment.

In this scenario, it is imperative to provide an unbiased definition of the brain areas or networks to be targeted with rt-fMRI-NF in pain conditions, and one promising direction is the use of rt-fMRI-NF informed by the targets employed by funcSurg approaches for chronic pain management. Against this background, we capitalized on funcSurg studies to identify critical hubs and networks as possible targets for the application of rt-fMRI-NF in the treatment of chronic pain.

## Materials and Methods

**Figure 1:**
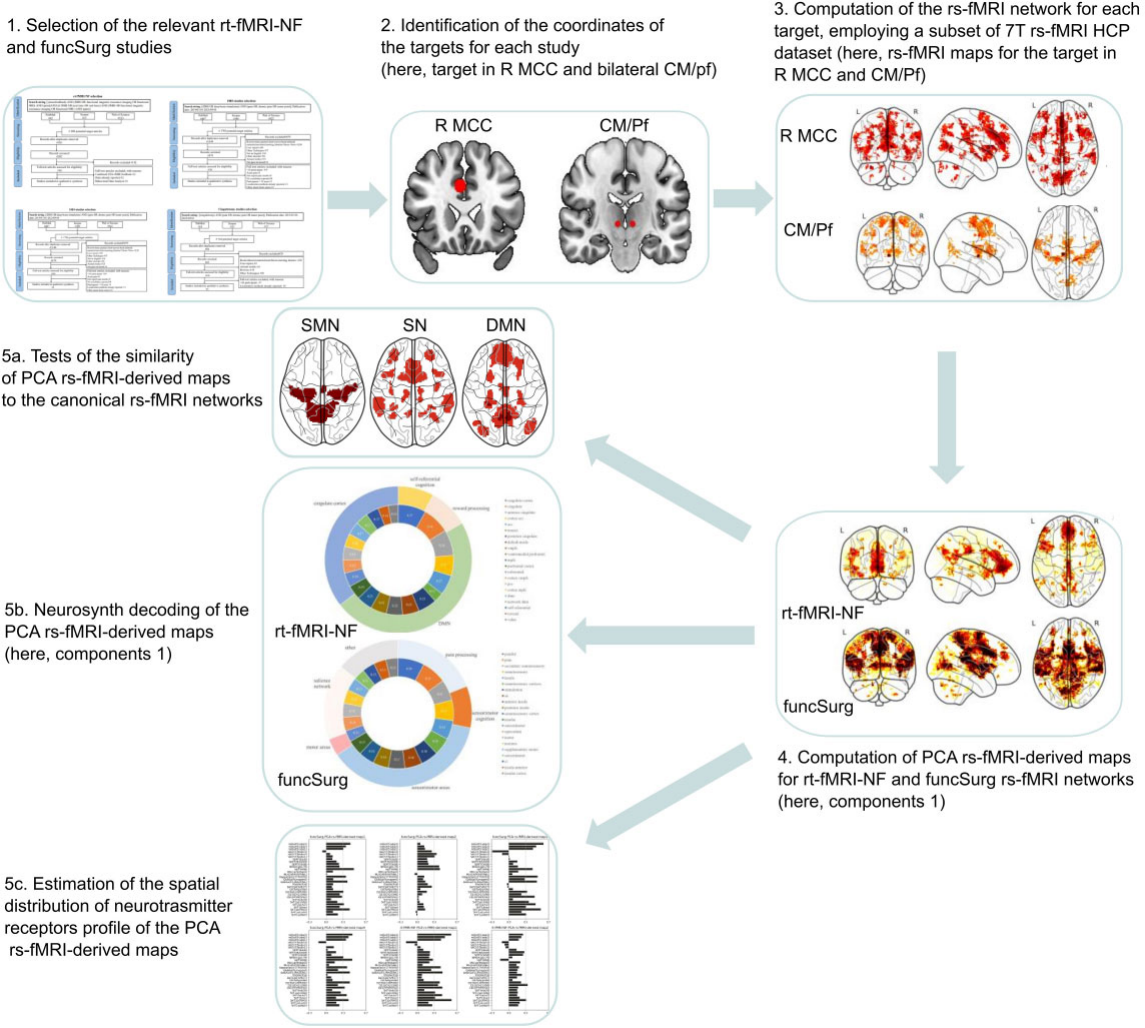
Overview of the analytical workflow.

Please refer to Fig. 1 for an overview of the analytical workflow.

### Records selection

#### rt-fMRI-NF records selection

The final literature search was conducted in August 2023 (starting from January 2000) and undertaken by one reviewer (L.D.) using the following strings: (“neurofeedback” OR “real-time”) AND (“fMRI” OR “functional magnetic resonance imaging” OR “functional MRI”) AND “pain” in Web of Science, PubMed, and Scopus. Records were screened by two independent reviewers (L.D. and Y.M.). From the selected papers, coordinates of the brain targets in the standardized space were collected. See the Supplementary Materials for additional information.

#### Functional Surgery Records Selection

To identify the targets of funcSurg approaches in chronic pain, we relied on the most recent meta-analyses in the field by selecting articles from the cited studies. For this purpose, we searched for meta-analyses published from January 2000 to August 2023 in Web of Science, PubMed, and Scopus with the following keywords: (DBS OR deep brain stimulation OR cingulotomy OR thalamotomy) AND (pain OR chronic pain OR tumor pain) AND (meta-analysis OR review). In our work, we did not consider studies employing motor cortex stimulation to treat pain because the use of electrodes covering a vast portion of the motor cortex prevented the identification of relatively precise localization (Lavrov *et al*., 2021). In addition, because rt-fMRI-NF applies only to gray matter areas, we excluded studies that targeted exclusively white matter (such as the posterior limb of the internal capsule) (Nguyen *et al*., 2011). Next, to identify recently published articles, we performed an additional literature search in the same databases from the publication date of the identified meta-analysis until August 2023 employing the following keywords: (DBS OR deep brain stimulation) AND (pain OR chronic pain OR tumor pain) for DBS; (cingulotomy) AND (pain OR chronic pain OR tumor pain) for cingulotomy; (thalamotomy OR MRgFUS OR Gamma Knife) AND (pain OR chronic pain OR tumor pain) for thalamotomy. In cases where recent meta-analyses were not available, such as in the case of thalamotomy, we performed the relevant literature search from January 2000 until August 2023. Based on the collected information relative to the localization of the target, three senior researchers (S.F., A.N., and L.M. in the acknowledgment note) independently computed the coordinates of each target in the Montreal Neurological Institute (MNI) space (see the Supplementary Materials for additional information).

### Identification of the rs-fMRI networks underlying the target regions

We identified the underlying rs-fMRI networks of all the identified targets, building 6 mm (for cortical targets) and 3 mm (for subcortical targets) radius regions of interest (ROI) centered in the identified MNI coordinates employing Marsbar (v.0.44). Using CONN toolbox v.22a (www.nitrc.org/projects/conn) (Nieto-Castanon, 2020), we calculated the seed-based functional connectivity of each ROI (as seed for rt-fMRI-NF) or a couple of ROI (for funcSurg) employing rs-fMRI data from 30 participants (age: M = 29.17 years, SD = 3.32 years; 20 females) of the Young Adult HCP dataset (Smith *et al*., 2013; Van Essen *et al*., 2013). For details, see the Supplementary Materials.

On the obtained thresholded rs-fMRI maps (eight maps for the rt-fMRI-NF dataset and eight maps for the funcSurg dataset), and separately for each dataset (rt-fMRI-NF and funcSurg), we employed principal component analysis (PCA) to identify the dominant patterns across the functional networks while minimizing data complexity. To implement this step, we used the PCA class from the scikit-learn library in Python (v.3.11.7). This algorithm performs linear dimensionality reduction through singular value decomposition, enabling the projection of data into a lower-dimensional space. PCA produces components that are orthogonal and normalized, ensuring each one represents a unique direction. These components are also ordered by the amount of variance they capture, with the first components reflecting the most significant variations in the data. It is a common practice in PCA to retain a predefined percentage of total variance to determine the number of principal components, with 70% often being used as a standard cut-off (Jolliffe and Cadima, 2016). Although this threshold is subjective, it serves as a widely accepted balance between model simplicity and accuracy. In this study, we chose an 80% threshold to ensure that a greater portion of the data’s variability was retained, even from less dominant rs-fMRI maps. This allowed that potentially significant information was not lost during dimensionality reduction. After this step, we generated new rs-fMRI maps (referred to as “PCA rs-fMRI-derived maps”) that embodied the core information captured by the selected principal components. In subsequent analyses, we assessed the degree to which these PCA rs-fMRI-derived maps overlapped with established rs-fMRI networks—the salience, sensorimotor, and default mode (DMN) networks (DMN)—hereafter called “canonical rs-fMRI networks” (Shirer *et al*., 2012). This allowed us to identify which canonical functional networks our PCA rs-fMRI-derived maps belonged to.

To this aim, we used a null map approach (Burt *et al*., 2020) as described in Ferraro *et al*. (2022). See the Supplementary Materials for further details.

### Neurosynth decoding of PCA rs-fMRI-derived maps

To identify the cognitive processes associated with the PCA rs-fMRI-derived maps, we performed the functional decoding employing the “decoder” function in Neurosynth (https://neurosynth.org/) (Yarkoni *et al*., 2011). This function decodes cognitive states from brain images by analyzing the spatial correlation between the map of interest and meta-analytic activation maps in its database. For each PCA rs-fMRI-derived map, the Neurosynth decoder produced a ranked list of terms and their corresponding correlation coefficients. For interpretation purposes, we retained the top 20 terms.

### Neurotransmitter receptors profiling of PCA rs-fMRI-derived maps

To estimate the spatial distribution of neurotransmitter receptors within each PCA rs-fMRI-derived map, we employed the JuSpace toolbox version 1.5 (available at https://github.com/juryxy/JuSpace (Dukart *et al*., 2021). This toolbox allowed the estimation of spatial correlations between our PCA rs-fMRI-derived maps and neurotransmitter receptor distribution maps obtained through nuclear imaging techniques These neurotransmitter receptors’ linearly rescaled maps are derived from different studies that report the average receptor maps observed in groups of healthy participants (see the Supplementary Materials for additional information).

### Neurosynth meta-analytic brain maps of “pain” and “chronic pain” terms

To have an unbiased representation of the brain activity during pain and chronic pain processing, we employed Neurosynth (https://neurosynth.org/) (Yarkoni *et al*., 2011) to obtain meta-analytic maps of the terms “pain” and “chronic pain.” More specifically, for each term, we obtained the uniformity test map that represents the uniform distribution of brain activity associated with that term, thus showing the extent to which each voxel is consistently activated in studies employing the specific word “pain” or “chronic pain.” Next, we ought to determine which canonical rs-fMRI networks the meta-analytic brain maps belong to. Also, in this case, we determined if the identified meta-analytic maps significantly overlapped with the selected canonical rs-fMRI maps (i.e. salience, DMN, and sensorimotor) employing a null map approach (Burt *et al*., 2020), as described in the previous section.

## Results

### Key findings

1. Identification of targets and membership of their PCA rs-fMRI-derived maps to canonical rs-fMRI networks:
  - rt-fMRI-NF targets were identified in the cingulate cortex and in the left insular cortex. The most representative component of the PCA rs-fMRI-derived maps (accounting for 76% of the total variance) belongs to the DMN and the salience network.
  - funcSurg targets were identified in bilateral MCC and thalamic nuclei (CM/pf, VPL/VPM, CLp). The four representative components of the PCA rs-fMRI-derived maps belong to the salience network, with three out of four maps also belonging to the sensorimotor network. No map belongs to the DMN.
2. Neurosynth functional decoding of the PCA rs-fMRI-derived maps:
  - rt-fMRI-NF targets: component 1 was linked with self-referential and reward processing, while component 2 was mainly associated with sensorimotor processes.
  - funcSurg targets: components reflected terms related to pain processing, sensorimotor cognition, and emotion.
3. Neurotransmitter receptor profiling of PCA rs-fMRI-derived maps:
  - rt-fMRI-NF targets: component 1 was significantly correlated with the FDOPA(f18) receptor map, while component 2 was correlated with the NAT receptor map.
  - funcSurg targets: three out of four components showed significant correlations with the NAT receptor map.
4. Neurosynth meta-analytic brain maps of terms “pain” and “chronic pain” and their membership to canonical rs-fMRI networks:
  Meta-analytic maps associated with “pain” and “chronic pain” mainly highlighted a network belonging to the salience network with no overlap with the DMN or sensorimotor network.

### Records selection

#### rt-fMRI-NF records selection

The literature search (flowchart of the screening process in Supplementary Fig. S1) resulted in 202 articles after removing the duplicates. Based on the title and the abstract, 192 articles were excluded, leaving 10 papers. After the entire reading, five papers were excluded. The remaining five records investigated a total of 84 healthy individuals (DeCharms *et al*., 2005; Emmert *et al*., 2014; Guan *et al*., 2015; Rance *et al*., 2014a,b) and 28 chronic pain patients (DeCharms *et al*., 2005; Guan *et al*., 2015). These studies have employed rt-fMRI-NF to evaluate the feasibility and the behavioral effects of modulating specific brain targets on laboratory-induced acute pain (DeCharms *et al*., 2005; Emmert *et al*., 2014; Rance *et al*., 2014a,b), and chronic pain perception (DeCharms *et al*., 2005; Guan *et al*., 2015). The MNI coordinates of the targets (original coordinates expressed in the TAL stereotactic space in DeCharms *et al*. (2005), Guan *et al*. (2015), and Rance *et al*. (2014a,b) were identified in the MCC (DeCharms *et al*., 2005), in the supracallosal (Emmert *et al*., 2014), subgenual (Rance *et al*., 2014a,b), and pregenual (Guan *et al*., 2015) anterior cingulate cortex (ACC), in the posterior insula cortex (posterior longitudinal gyrus) (Rance *et al*., 2014a,b), and in the anterior insula cortex (aIns) (middle short gyrus) (Emmert *et al*., 2014). All the studies reported that the participants were able to learn to modulate the activity of the target. Two of them, investigating the modulation of acute pain in healthy participants and chronic pain in chronic pain patients, also reported a decrease in the level of pain (DeCharms *et al*., 2005; Guan *et al*., 2015). In particular, DeCharms *et al*. (2005) used rt-fMRI-NF to study how the willful modulation of MCC affects acute pain and chronic pain. Testing eight healthy participants and four control groups under different conditions, they observed that only the experimental group learned to modulate the activity of the MCC and concomitantly reported a reduction in the level of perceived acute pain. In the same study, they investigated 12 patients (eight experimental and four control) with chronic pain, observing that the regulation of MCC activity reduced the level of perceived chronic pain. However, a subsequent study by the same group on a large sample did not replicate these results (Guan *et al*., 2015). Guan *et al*. (2015), in a double-blinded randomized study, showed that six out of eight postherpetic neuralgia patients learned to regulate pregenual ACC activity with significant pain reduction in comparison to a sham group. In this case, acute pain was used to identify the pregenual ACC.

See the identified coordinates in Table 1 and specific information in the Supplementary Materials and Table S1. The selected studies are described in the Supplementary Materials.

**Table 1:** Extracted coordinates of the rt-fMRI-NF targets

Author	Target area (as reported)	Target area (AAL3)	ROI radius (mm)	MNI coordinates		
				*x*	*y*	*z*
DeCharms *et al*. (2005)	R rostral ACC	R MCC	6	4	22	30
Emmert *et al*. (2014)	L aIns	L aIns	6	−38	4	3
	L ACC	L ACC_sup	6	−1	21	26
Rance *et al*. (2014a)	rostral ACC	L ACC_sub	6	0	37	−2
	L pIns	L pIns	6	−42	−19	11
Rance *et al*. (2014b)	L rostral ACC	L ACC_sub	6	−3	35	−1
	L pInsl	L pInsl	6	−45	−4	−7
Guan *et al*. (2015)	R rostral ACC	R ACC_pre	6	1	41	1

Abbreviations: ACC_sub, subgenual ACC; ACC_pre, pregenual ACC; ACC_sup, supracallosal ACC; pIns, posterior insula. To localize the regions, we employed and used AAL3 (Rolls *et al*., 2020) for all the other regions.

## Functional Surgery Records Selection

See the identified coordinates for the targets in Table 2 and specific information in the Supplementary Materials and Table S2. The coordinates calculated by reviewers 2 (L.M.) and 3 (A.N.) were <5 mm from the coordinates of reviewer 1 (S.F.) (mean 2.5 mm, range 0–5 mm).

**Table 2:** Coordinates of the funcSurg studies

Author	Region	ROI radius (mm)	MNI coordinates					
			Left			Right		
Yen *et al*. (2005)	MCC	6	−6	5	34	6	5	34
Strauss *et al*. (2018)	MCC	6	−8	9	33	8	9	33
Boccard *et al*. 2017)	MCC	6	−7	9	31	7	9	31
Urgosik *et al*. (2018)	CM/pf	3	−8	−19	0	8	−19	0
Lovo *et al*. (2019)	CM/pf	3	−8	−22	3	8	−22	3
Abdallat *et al*. (2021)	CM/pf	3	−8	−20	−1	8	−20	−1
Abreu *et al*. (2017)	VPL/VPM	3	−19	−21	6	18	−20	6
Rasche *et al*. (2006)	VPL/VPM	3						
Abdallat *et al*. (2021)	VPL/VPM	3						
Gallay *et al*. (2023)	CLp	3	−11	−25	6	11	−25	6

## Deep brain stimulation (DBS)

Based on the results of the meta-analyses (see in Supplementary Fig. S2) for DBS, we capitalized on the recent work by Frizon *et al*. (2020), which identified 12 studies. From these papers, three were excluded because of the sample size (Hollingworth *et al*., 2017; Hunsche *et al*., 2013; Lempka *et al*., 2017), two since they did not sufficiently characterize the target (Coffey, 2001; Yamamoto *et al*., 2006), and one (Hamani *et al*., 2006) because no successful effect on pain was found. Of the remaining six studies, three from the same group (Abreu *et al*., 2017; Boccard *et al*., 2013; Pereira *et al*., 2013) reported the same localization of the target in the thalamic nuclei; the other two papers also from the same group (Boccard *et al*., 2014, 2017) localized the target in the cingulate cortex. In agreement with the AAL3 atlas, this region was the MCC, although the authors referred to it as the ACC in accordance with the different nomenclature. For all these papers reporting the same target location, the target was identified through the description found in Abreu *et al*. (2017) for VPL/VPM and in Boccard *et al*. (2017) for the MCC. Thus, from the remaining three papers, we extracted the localization of targets located in VPL/VPM (Abreu *et al*., 2017; Rasche *et al*., 2006) and in the cingulate cortex (Boccard *et al*., 2017). The search for more recent papers (see Supplementary Fig. S3) with the selected criteria identified the Abdallat *et al*. (2021) study targeting the CM/Pf and VPL/VPM and Abreu *et al*. (2022) that, however, reported a follow-up of a previous study and thus was excluded. Since the computed MNI coordinates of VPM/VPL in Abdallat *et al*. (2021), Abreu *et al*. (2022), and Rasche *et al*. (2006) did not appear to be at the expected position when superimposed on the thalamic atlas in the MNI space (Saranathan *et al*., 2021), we relied on the center of mass of VPL/VPM extracted from the same atlas. Notably, we selected only one paper, also targeting the coordinates of VPL/VPM, reporting the coordinates of the PAG/PVG (Rasche *et al*., 2006). However, since the extracted PAG/PVG coordinates appeared not to be in the expected position, they were not selected for the successive analyses.

## Cingulotomy

In the selected review (Sharim and Pouratian, 2016), 11 studies were reported; however, eight were published before 2000. This left two studies from the same group (Yen *et al*., 2005, 2009) reporting the same target localization and one study (Patel *et al*., 2015) on three patients that was therefore discarded. We also searched for more recent papers from January 2016 to August 2023 (see Suppplementary Fig. S4) and we found a study by Wang *et al*. (2017) employing the same target localization as reported by Yen *et al*. (2005); Strauss *et al*. (2018) investigating a slightly different localization of the target; and Hochberg *et al*. (2020) using the same target localization as reported by Strauss *et al*. (2018). So, for the localization of the cingulate target, we relied on the papers by Yen *et al*. (2005) and Strauss *et al*. (2018).

## Thalamotomy

The literature search (see Supplementary Fig. S5), according to the selected criteria, identified five papers targeting the posterior central lateral thalamic nuclei (CLp) (Gallay *et al*., 2020, 2023; Jeanmonod *et al*., 2001, 2012) and the CM/Pf complex (Lovo *et al*., 2019; Urgosik and Liscak, 2018). Notably, for the CLp, several findings were reported by the same group (Gallay *et al*., 2023; Jeanmonod *et al*., 2001, 2012), with the target being updated in their most recent paper. Thus, we considered only the most recent paper (Gallay *et al*., 2023).

### Identification of the rs-fMRI networks underlying the target regions

**Figure 2:**
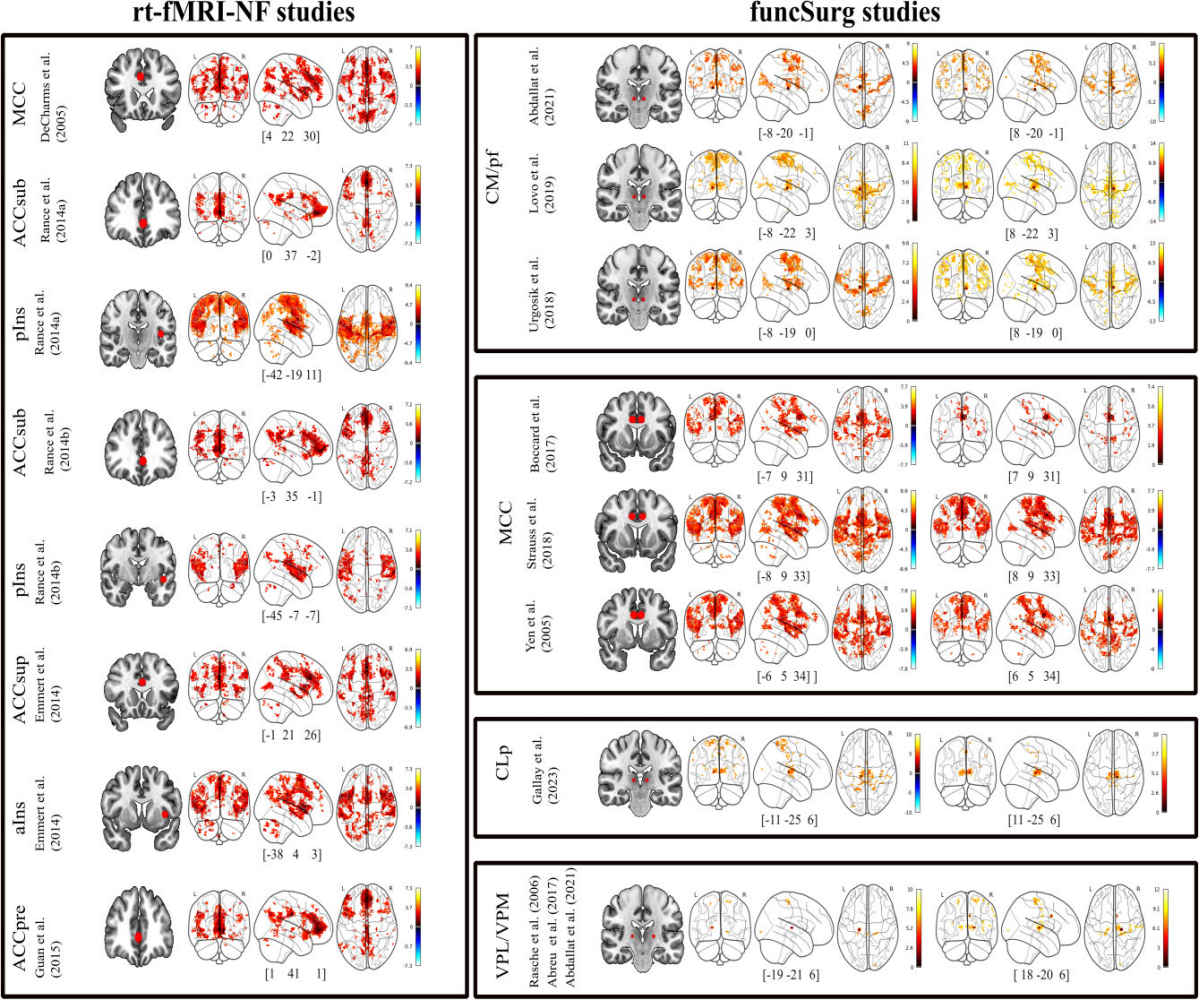
Glass-brain representations of the rs-fMRI maps obtained from each seed computed as a 6 or 3 mm sphere (see the main text for details) centered in the coordinates of the identified studies' region of interest. Abbreviations: ACCsub, subgenual ACC; ACCsup, supracallosal ACC; ACCpre, pregenual ACC; pIns, posterior insula.

Based on the identified MNI coordinates, eight ROI for the rt-fMRI-NF records (eight targets identified in right MCC, left aIns, left pIns, and left pregenual, subgenual, and supracallosal ACC), and eight bilateral ROI (targets in CLp, CM/Pf, VPL/VPM, and MCC) for funcSurg records were built to produce functional connectivity maps (or SBC maps) on the 7 T MRI HCP dataset (see Fig. 2).

**Figure 3:**
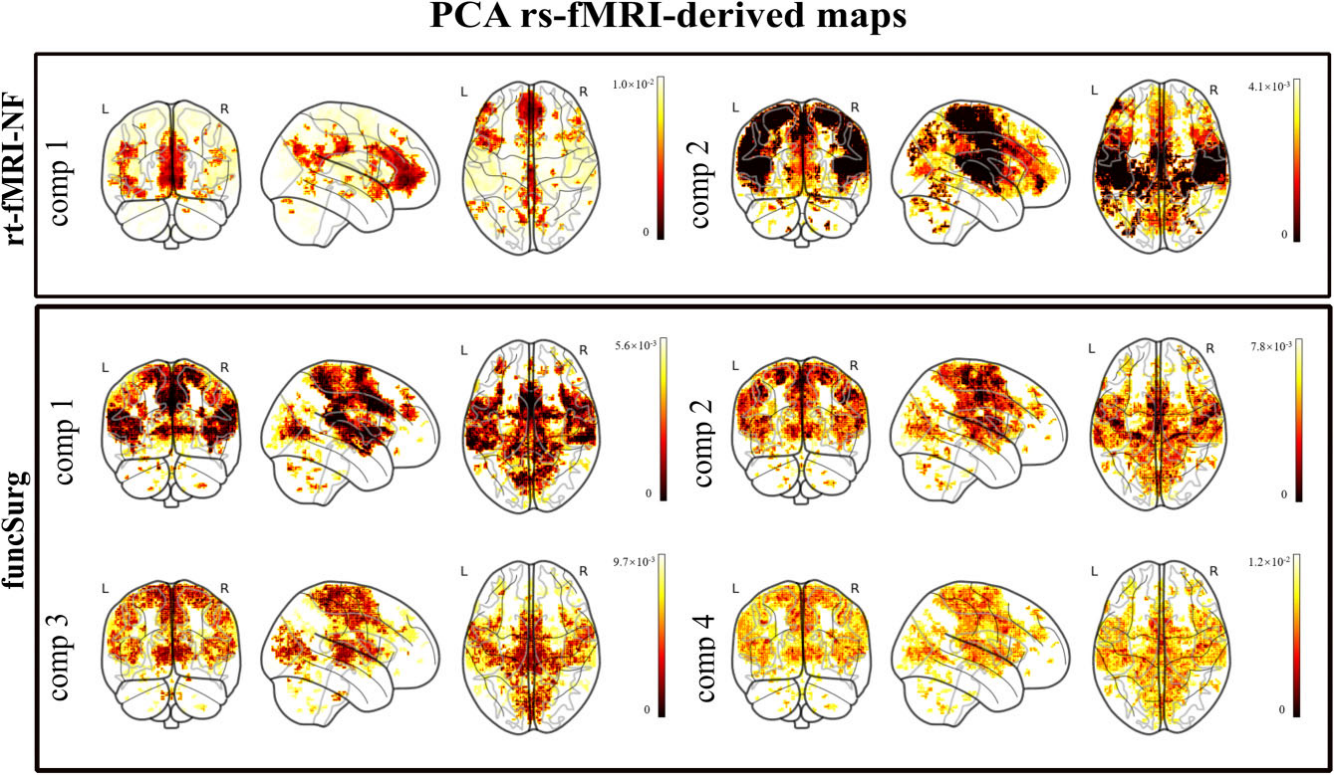
PCA rs-fMRI-derived maps. Abbreviation: comp, component.

The PCA applied to the HCP rs-fMRI maps obtained from the rt-fMRI-NF targets (see Fig. 3) identified two components, accounting for a cumulative explained variance of 84.7%. For the rt-fMRI-NF ROI, component 1 accounted for 76% of the total variance, indicating that a substantial portion of the neural activity across the rt-fMRI-NF ROI rs-fMRI HCP maps is captured by a single neural network. This bilateral network includes the cingulate and insula cortex. Component 2, accounting for 8.7% of the total variance, showed prominent bilateral clusters in the MCC, in sensorimotor areas, and in the insula cortex.

Regarding the PCA performed on the HCP rs-fMRI maps from the funcSurg targets (see Fig. 3), four components were detected with a cumulative explained variance of 83%. Component 1, explaining 37% of the variance, encompasses a bilateral network comprising the insula cortex, the MCC, and the sensorimotor areas. Component 2, accounting for 20.4% of the variance, was characterized by a bilateral network comprising the MCC, parietal regions, and areas of the insula cortex. Component 3, accounting for 13.6% of the total explained variance, comprised mainly bilateral sensorimotor areas and MCC with large clusters in the thalamus. Component 4 (12% of the total explained variance) was characterized by a bilateral network comprising the sensorimotor areas, the MCC, and clusters in the insula cortex covering mainly the posterior regions (see Supplementary Table S3 for details).

Formal tests of the similarity of the PCA rs-fMRI-derived maps to the canonical rs-fMRI networks (DMN, salience network, and sensorimotor network) showed that the rt-fMRI-NF components 1 and 2 significantly overlapped with the DMN. Additionally, component 1 overlapped, although modestly, with the salience network, while component 2 overlapped with the sensorimotor network. All the selected funcSurg components were significantly similar to the salience network. Additionally, components 1, 3, and 4 also significantly overlapped with the sensorimotor network (see Table 3).

**Table 3:** Formal tests of the similarity of the PCA rs-fMRI-derived maps to the canonical rs-fMRI networks (salience network, DMN, sensorimotor network).

Components	SN		DMN		SMN	
	*P* value	emp	*P* value	emp	*P* value	emp
rt-fMRI-NF PCA comp1	0.006*	0.040	0.001*	0.149	0.999	0.000
rt-fMRI-NF PCA comp2	0.103	0.078	0.002*	0.098	0.001*	0.289
funcSurg PCA comp1	0.001*	0.417	0.964	0.054	0.023*	0.127
funcSurg PCA comp2	0.001*	0.142	0.874	0.023	0.946	0.016
funcSurg PCA comp3	0.001*	0.148	1.000	0.156	0.001*	0.138
funcSurg PCA comp4	0.001*	0.150	0.999	0.026	0.001*	0.204
NS: term ‘chronic pain’	0.001*	0.105	0.995	0.008	0.997	0.003
NS: term ‘pain’	0.001*	0.490	0.928	0.069	0.994	0.037

Abbreviations: emp, empirical value; SN, salience network; SMN, sensorimotor network; comp; rt-fMRI-NF PCA: PCA rs-fMRI-derived map of real-time fMRI neurofeedback targets; funcSurg PCA: PCA rs-fMRI-derived map of funcSurg targets; comp, component; NS: Neurosynth meta-analytic map. *P < 0.05.

### Neurosynth decoding of PCA rs-fMRI-derived maps

**Figure 4:**
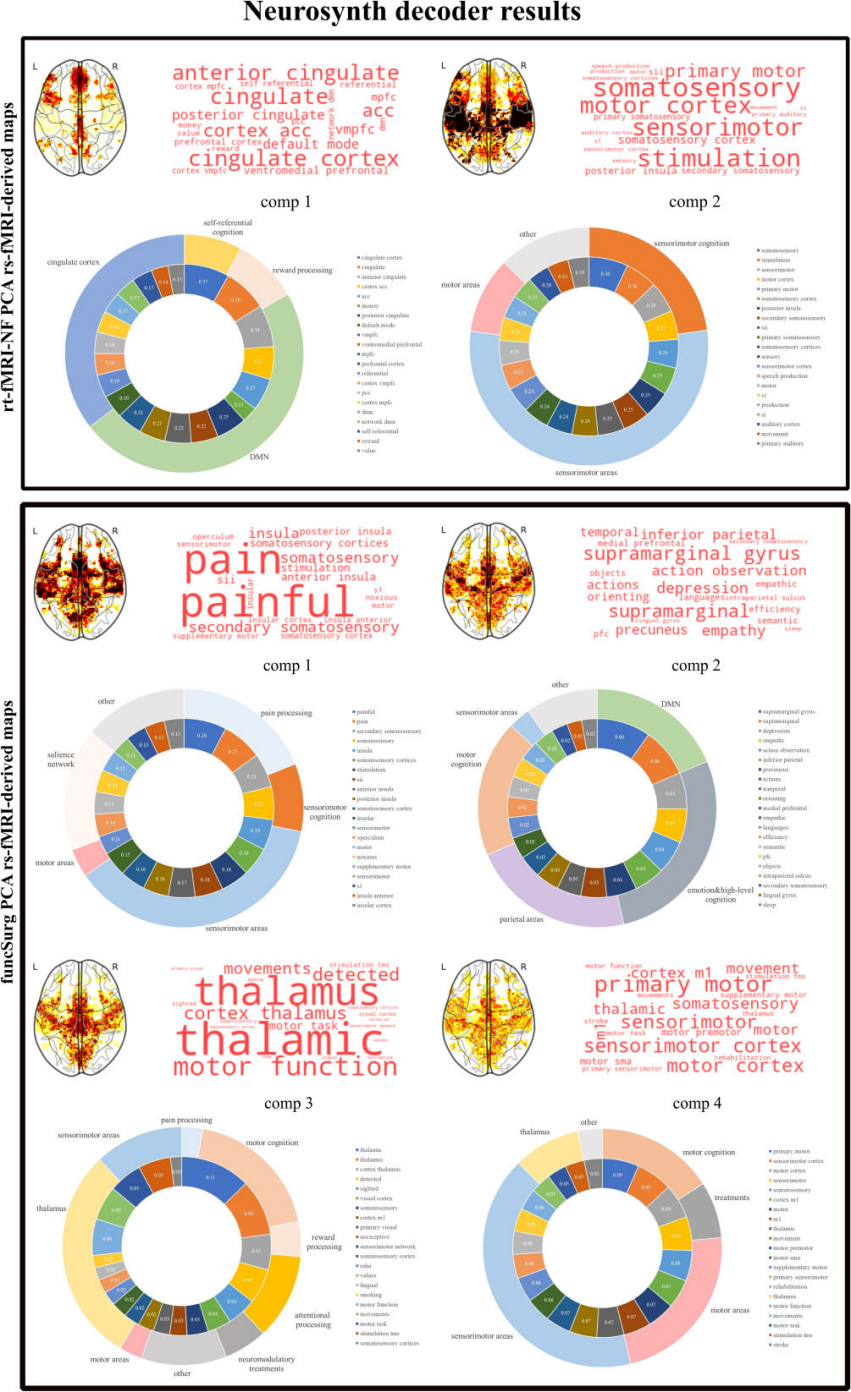
Neurosynth decoding of PCA rs-fMRI-derived maps retaining the top 20 terms (correlation values in the inner circle). Abbreviation: comp, component.

For a detailed view of the decoding analyses, please refer to Fig. 4 and Supplementary Table S4. In summary, the Neurosynth decoding of the rt-fMRI-NF PCA rs-fMRI-derived maps highlighted that component 1 was associated with self-referential and reward processing and anatomical terms for the DMN and ACC, whereas component 2 was linked mainly to sensorimotor functions and the motor cortex. For funcSurg PCA-derived maps, component 1 primarily correlated with pain processing and sensorimotor cognition, aligning with the sensorimotor and salience networks. Component 2 connected sensorimotor and emotional cognition to parietal and DMN regions, while components 3 and 4 included associations with reward, attention, and neuromodulatory treatments, largely within the sensorimotor, visual cortex, and thalamic areas.

### Neurotransmitter receptors profiling of PCA rs-fMRI-derived maps

Spatial correlation analysis between each PCA rs-fMRI-derived map and the neurotransmitter receptor distribution maps showed that, in the case of the rt-fMRI-NF targets, component 1 was significantly correlated with the FDOPA(f18) receptor map, and component 2 with the NAT receptor map. Regarding funcSurg targets, spatial correlation analysis revealed consistent patterns of significant correlations, with three out of four components showing significant correlations with the NAT receptor map (see Supplementary Fig. S6 and Table S6).

### Neurosynth meta-analytic brain maps of “pain” and “chronic pain terms”

Neurosynth uniformity maps indicated that “chronic pain” was linked to robust clusters in regions including the insula, inferior parietal cortex, and amygdala, extending to the MCC and ACC, among others. The more extensive “pain” uniformity map showed that this term was mainly associated with activity in the bilateral insula, pregenual cingulate cortex, and subgenual regions of the frontal and motor areas (supplementary and pre-supplementary). The formal test employing null maps with preserved spatial autocorrelation showed that these two maps significantly overlapped with the salience network but not with the DMN nor the sensorimotor network (see Supplementary Table S5).

## Discussion

FuncSurg approaches for the treatment of chronic pain are considered highly efficacious but are not routinely applied in clinical practice primarily because of their invasiveness. However, they provide highly specific and potentially causal manipulations for informing target regions for techniques involving non-invasive neuromodulation targeting chronic pain, including rt-fMRI-NF. Despite the remarkable potential of rt-fMRI-NF in the treatment of pain results remained inconsistent and our systematic review aimed to characterize the rt-fMRI-NF brain targets used in the current literature and to identify new ones, taking advantage of funcSurg studies that have shown modulation or lesioning of specific brain regions can effectively mitigate chronic pain. To facilitate a comprehensive overview, after identifying the brain targets of rt-fMRI-NF and funcSurg studies, we characterized, using several unbiased approaches, the underlying functional networks from neurofunctional, meta-analytic, and neurotransmitter perspectives.

We report three main findings. First, and as expected, we showed that the targets employed by rt-fMRI-NF are different from those used in funcSurg studies, except for the MCC, employed in several funcSurg studies considered here (Boccard *et al*., 2017; Strauss *et al*., 2018; Yen *et al*., 2005) and in only one rt-fMRI-NF study (DeCharms *et al*., 2005).

Second, we showed that rt-fMRI-NF targets are major hubs of a single functional circuit (component 1 of the PCA rs-fMRI-derived maps) that explains >70% of the variance in the target rs-fMRI maps and that, according to the large-scale meta-analytic “decoding” using Neurosynth, mainly maps onto regions typically assigned to the DMN. This was confirmed by comparing the overlap of this target map with the canonical rs-fMRI networks from an independent dataset (Shirer *et al*., 2012) that, however, also suggested an involvement of the salience network. By contrast, all funcSurg targets, although with some differences, are hubs of the sensorimotor network (components 1, 3, and 4 of the PCA rs-fMRI-derived maps) and of regions involved in motor cognition (components 1, 2, 3, and 4), as shown by the meta-analytic “decoding” with Neurosynth. The membership of these functional networks to the sensorimotor network was confirmed by comparing the overlap of the target maps with the canonical rs-fMRI networks (Shirer *et al*., 2012), which, however, also suggested a robust overlap with the salience network.

Third, we showed that the main rs-fMRI network associated rt-fMRI-NF targets (i.e. component 1 of the PCA rs-fMRI-derived maps) mainly converges on the FDOPA(f18) receptor map, while the rs-fMRI networks associated with funcSurg targets converge, except component 3, on the noradrenergic system (i.e. NAT receptor map).

The meta-analysis on rt-fMRI-NF in acute and chronic pain revealed only five studies that examined healthy individuals (DeCharms *et al*., 2005; Emmert *et al*., 2014; Guan *et al*., 2015; Rance *et al*., 2014a,b) and chronic pain patients (DeCharms *et al*., 2005; Guan *et al*., 2015). These studies targeted different areas of the ACC (rostral, subgenual, pregenual, and supracallosal ACC) (Emmert *et al*., 2014; Guan *et al*., 2015; Rance *et al*., 2014a,b), the MCC (DeCharms *et al*., 2005), and of the insular cortex (Emmert *et al*., 2014; Rance *et al*., 2014a,b). In general, participants were able to learn to modulate the target regions, but with conflicting results at the behavioral level. Studies involving healthy participants (Emmert *et al*., 2014; Rance *et al*., 2014a,b), with the exception of DeCharms *et al*. (2005), showed no significant behavioral changes in pain levels during acute pain infliction. In this regard, it is worth noting that although in Emmert *et al*. (2014) the modulation of the targets (left sup ACC and left aIns) resulted in a reduction in acute pain perception, the lack of a control condition and the fact that both regulators and non-regulators showed a similar behavioral response do not allow robust conclusions. Only DeCharms *et al*. (2005), targeting MCC, reported a reduction in acute pain in healthy participants and, in the same study, a decrease in the level of chronic pain in patients. However, these results were not replicated in a subsequent study of the same group (Hawkinson *et al*., 2012). Importantly, in chronic pain conditions, Guan *et al*. (2015) showed, in a double-blind randomized study, that the rt-fMRI-NF targeting of the pregenual ACC induced a reduction of the level of pain in six out of eight postherpetic neuralgia patients. The meta-analysis on funcSurg studies applied to chronic pain identified nine records that demonstrated a reduction of chronic pain in at least 40% of patients (Abdallat *et al*., 2021; Boccard *et al*., 2017; Gallay *et al*., 2023; Lovo *et al*., 2019; Rasche *et al*., 2006; Strauss *et al*., 2018; Urgosik and Liscak, 2018; Yen *et al*., 2005). From these investigations, eight bilateral targets were identified, specifically in the MCC (Boccard *et al*., 2017; Strauss *et al*., 2018; Yen *et al*., 2005) and in selected thalamic nuclei, namely VPL/VPM (Abdallat *et al*., 2021; Abreu *et al*., 2017; Rasche *et al*., 2006), CM/pf (Abdallat *et al*., 2021; Lovo *et al*., 2019; Urgosik and Liscak, 2018), and CLp (Gallay *et al*., 2023).

It is worth noting that both the rt-fMRI-NF studies in chronic pain and the funcSurg approaches mainly investigated the effects of these treatments in neuropathic pain, although, of course, the funcSurg studies have treated patients in very severe conditions. Interestingly, despite the similar diagnosis of the involved patients, these observations show that the anatomical targets of the rt-fMRI-NF and funcSurg approaches are quite different. Indeed, while the rt-fMRI-NF studies favored only cortical targets (particularly in the cingulate and insular area), the selected funcSurg approaches favored the thalamic nuclei and a single cortical target (in the MCC). These differences clearly lie in the diverse foundations of these approaches. In this regard, rt-fMRI-NF studies are rooted in the relatively recent fMRI investigations of acute pain, identifying their targets in areas that respond to nociceptive stimulation, particularly in areas primarily involved in processing the emotional and cognitive aspects of pain. Although this may be a pragmatic approach and may reflect the slippage of chronic pain (as opposed to acute pain) toward the known alterations in areas involved in emotion processing (Kuner and Kuner, 2021; Serafini *et al*., 2020), the effectiveness of this method in defining brain areas that can modulate chronic pain remains to be fully understood. Today, we have ample evidence indicating that chronic pain is different from acute pain (Martucci *et al*., 2014) and represents a distinct nosological entity with important neuroplastic reorganizations (Serafini *et al*., 2020). Furthermore, new possible treatments to reduce chronic pain should target antinociceptive networks (Price *et al*., 2018), which, however, are still not well-defined at the forebrain and midbrain levels.

Differently, functSurg approaches to treat chronic pain are deeply rooted in a long tradition of neurosurgical and neurophysiological studies (Jeanmonod and Morel, 2009). The medial thalamus, in particular, plays a significant role in the neurosurgical treatment of chronic pain. This thalamic region, which includes some of the included funcSurg targets (i.e. CLp and CM/pf), is conceptualized as part of the medial pain system (Bushnell *et al*., 2013; De Ridder and Vanneste, 2016; Kulkarni *et al*., 2005; Price, 2000) and due to its afferent (from the spinothalamic and spino-reticular thalamic tracts) and efferent (to the aIns and dorsal ACC/MCC) connections, mainly overlapping with the salience network, it is considered to be involved in the motivational-affective aspects of pain (De Ridder *et al*., 2021). VPL/VPM nuclei, according to the traditional view of the pathways of pain processing, are part of the lateral pain pathway (De Ridder and Vanneste, 2016; Kulkarni *et al*., 2005; Price, 2000), which maps onto the somatosensory cortex extending to the parietal area, and which mainly processes the discriminative/sensory components of pain (Bushnell *et al*., 2013; Flor *et al*., 1995; Kulkarni *et al*., 2005). As for the MCC, first pioneered in 1948 (Whitty *et al*., 1952), the neurosurgical lesioning of this region (described as ACC in these studies) has been performed to relieve intractable pain, particularly terminal cancer pain (Allam *et al*., 2022). The apparent effects of cingulotomy not so much on nociceptive pain but on the suffering associated with it set the stage for recent approaches to both lesion and modulation of this brain region. MCC is considered a critical structure processing the affective-cognitive components of pain.

Although the targets of the rt-fMRI-NF and funcSurg approaches are different, except for the MCC, it is possible that they could be different hubs within the same functional networks.

Our results for the rs-fMRI-NF targets showed that only two components explained most of the variance of the underlying functional networks. Notably, the first component alone explained >74% of the variance, indicating that most targets mainly probe a single extended functional network that, as demonstrated by large-scale meta-analytic decoding with Neurosynth (Yarkoni *et al*., 2011), maps mainly to regions involved in self-referential activity and reward processing, with robust anatomical overlap with areas of the DMN. The more lenient approach of evaluating the overlap with canonical networks from an independent rs-fMRI dataset (Shirer *et al*., 2012) confirmed that this extended network mapped onto the DMN but also, although modestly, onto the salience network.

The results for the funcSurg targets showed that most of the variance present in the underlying functional networks was explained by four components; in this case, the variance was more dispersed (from 12 to 37%). Here, the large-scale meta-analytic decoding with Neurosynth showed that all four rs-fMRI-derived PCA networks mapped robustly onto areas involved in sensorimotor cognition (components 1, 2, 3, and 4) and onto the sensorimotor areas (components 1, 3, and 4), with preferential anatomical overlap with regions of the motor system, albeit to varying degrees. The more lenient approach evaluating the overlap with the canonical networks from an independent rs-fMRI dataset (Shirer *et al*., 2012) supported this conclusion but also indicated that all these networks significantly overlap with the salience network.

Based on the previous premise, overall, these results indicate that the targets of rt-fMRI-NF studies informed by the funcSurg approaches should be sought mainly among the main hubs of the sensorimotor network, possibly in the motor system and among regions of the salience network, such as the aIns and the MCC already targeted by the previous rt-fMRI-NF studies. In this context, it is important to emphasize that in rt-fMRI-NF studies with the goal of controlling acute pain, targeting the areas of the functional networks identified from funcSurg studies designed for chronic pain may not be appropriate. By contrast, when focusing on chronic pain, it is essential to target ROI specifically associated with the control of this type of pain. In this context, a functional localizer using acute pain stimuli to identify the target area might be less effective, whereas a resting-state functional network localizer with the intent to identify the hubs of the networks observed in this work might be a more suitable choice. It is important to note that rt-fMRI-NF provides flexibility in its application, as it can be used not only to upregulate or downregulate (Paret *et al*., 2018; Rance *et al*., 2014b) specific target regions but also to modulate the functional connectivity between regions of interest (Zhao *et al*., 2019). This adaptability allows rt-fMRI-NF to be tailored to diverse therapeutic goals, enhancing its potential effectiveness in various contexts.

It is important to emphasize that the motor cortex is a key target of invasive (motor cortex stimulation) and non-invasive (transcranial magnetic stimulation or direct transcranial stimulation) approaches to controlling chronic pain. Although further studies are needed to assess its efficacy, a recent clinical study has shown that (invasive) stimulation of the motor cortex can reduce the level of pain in a considerable number of neuropathic pain patients (Hamani *et al*., 2021). Similarly, non-invasive stimulation methods on primary motor areas can induce analgesic effects, perhaps through the restoration of defective endogenous pain inhibitory pathways (Giannoni-Luza *et al*., 2020); however, their effects appear to be short-lived (Nguyen *et al*., 2011).

Although less supported by our results, since it emerges only with the canonical rs-fMRI networks approach, the salience network can also be considered a target of the rt-fMRI-fMRI. The salience network, anchored in the aIns and the MCC (Seeley *et al*., 2007) plays an important role in the guidance of flexible behavior and detecting salient events in the environment (Menon and Uddin, 2010; Seeley *et al*., 2007; Yao *et al*., 2018) integrating the current interoceptive state and autonomic feedback with internal goals and external demands (Craig, 2009; Yao *et al*., 2018). Recently, the salience network was shown to robustly overlap with the central autonomic network (Ferraro *et al*., 2022). Moreover, abnormalities of the salience network have been repeatedly observed in chronic pain, with a recent meta-analysis (Ferraro *et al*., 2021) showing robust dysregulations of the left aIns in these conditions.

Checking whether the targets of the rt-fMRI-NF and funcSurg studies engaged the same or different canonical rs-fMRI networks, rather than conducting direct exploratory comparisons between the functional maps of the two approaches, allowed us to interpret our results within a robust theoretical framework and to gain important insights in respect to the possible new rt-fMRI-NF targets. Importantly, canonical rs-fMRI networks are not only very well documented in the literature, but also exhibit highly conserved topography even under pathological conditions, despite potential changes in connectivity metrics (e.g. AALF, ReHo). These two features provide the basis for robust interpretation of results even in the context of chronic pain conditions. Furthermore, it is important to note that most funcSurg targets for chronic pain are relatively small subcortical nuclei (in thalamic and midbrain), which pose serious challenges for their modulation by rt-fMRI-NF because of their size and low signal-to-noise ratio. Our approach, therefore, allowed us to identify canonical rs-fMRI networks involved in chronic pain control amenable to neuromodulation with rt-fMRI-NF through their main cortical hubs. In addition, these identified canonical networks provide reliable references for localizing functional hubs to be modulated in rt-fMRI-NF studies. These networks can be identifiable at the single-participant level with relatively short scan times, offering a practical alternative for target localization without requiring specific fMRI tasks, such as those involving the use of acute pain stimulation, which we consider, in principle, potentially inadequate for identifying areas to be modulated in chronic pain conditions.

Interestingly, the uniform map obtained from Neurosynth for the term “chronic pain” (along with the term “pain”) robustly overlaps with regions of the salience network but not with the sensorimotor network. Interestingly, the uniform map obtained by Neurosynth for the term “chronic pain” (along with the term “pain”) overlaps robustly with regions of the salience network but not with regions of the sensorimotor network. This intriguing dissociation between the neural processing of chronic pain and the antinociceptive effects induced by the modulation of funcSurg targets (mapping onto the sensorimotor network and less robustly to the salience network) suggests that the neural pathways of these linked processes might be fundamentally different.

Notably, closely related to the observation that all functional networks underlying funcSurg targets map onto the sensorimotor and salience network, here we have also provided robust evidence that, despite a different involvement of additional neurotransmitter systems, three out of four networks underlying funcSurg targets map onto the noradrenergic system. This observation suggests that the funcSurg approaches here investigated can regulate the noradrenergic system.

Our results on the neurotransmitter profiling further emphasize a unique role of the noradrenergic system in funcSurg targets, which could underlie its effects on chronic pain's vigilance and attentional aspects. Noradrenergic pathways are known to play dual roles in pain modulation—both antinociceptive and pronociceptive, depending on the network state—highlighting the complex interplay of neurotransmitter systems in chronic pain (Taylor and Westlund, 2017).

It is worth noting that various meta-analyses (Bittar *et al*., 2005; Frizon *et al*., 2020) have shown that DBS is able to successfully treat nociceptive pain (e.g. intractable low back pain) and, among neuropathic pain types, peripheral neuropathic pain. Therefore, future rt-fMRI-NF studies should take these findings into account, potentially focusing on these specific pain types to optimize therapeutic outcomes.

## Limitations

Despite interesting results, this work is clearly limited. First of all, the extrapolation of coordinates from funcSurg studies, which did not report the exact coordinates in MNI space, was a major challenge. To overcome this issue, three senior experts in neuroimaging data (S.F., A.N., L.M.) independently extracted the coordinates, and subsequently, after the verification that no identified coordinate varied by >5 mm, the center of mass was computed, and it was cross-referenced with the *in vivo* high-resolution structural MRI human thalamic atlas (Saranathan *et al*., 2021). Second, we relied heavily on rs-fMRI data, inherently characterized by low spatial resolution, to identify the underlying rs-fMRI networks. However, in an attempt to mitigate this limitation, we utilized the high-resolution neuroimaging HCP dataset obtained at 7-T MRI (spatial resolution of 1.6 mm isotropic voxels). In addition, it is worth noting that the spatial extent of activation in thalamic DBS, with a stimulation amplitude ranging from 1 to 3.5 V, typically results in an average effective activation radius of 2.0 to 3.9 mm. This falls within the range of our chosen ROI size of 3 mm, underscoring the compatibility of the DBS activation radius with our ROI dimensions (Kuncel *et al*., 2008). Furthermore, the clear emergence of four distinct PCA rs-fMRI-derived maps for the funcSurg targets supports our findings, indicating differences among the different targets. As a note of caution, because the funcSurg studies considered here mainly investigated patients with neuropathic pain, we do not know the extent to which these results apply to patients suffering from other types of chronic pain.

## Conclusions

Informed by the long tradition of funcSurg studies, our meta-analytic approach robustly supports the hypothesis that rt-fMRI-NF targets should be identified in areas of the motor system and into areas of the salience network. Since areas of the salience network have already been investigated with inconsistent findings, future studies should investigate with rigorous clinical trials whether the motor system is a suitable target for the rt-fMRI-NF for the treatment of chronic pain.

## Role of the Funder/sponsor

The funders had no role in the design and conduct of the study; collection, management, analysis, and interpretation of the data; preparation, review, or approval of the manuscript; and decision to submit the manuscript for publication. Any opinions, findings, conclusions, or recommendations expressed in this publication do not reflect the views of the Government of the Hong Kong Special Administrative Region or the Innovation and Technology Commission.

## Ethics approval and consent to participate

Not applicable.

## Supplementary Material

kkae026_Supplemental_File

## Data Availability

Data files are available in the OSF directory: https://osf.io/cmgvq/
