# Supplementary material for: Identifying brain targets for real-time fMRI neurofeedback in chronic pain: insights from functional neurosurgery
Source: Psychoradiology. 2024 Nov 21;4:kkae026. doi: 10.1093/psyrad/kkae026 (PMC11683833; doi:10.1093/psyrad/kkae026)

Supplementary Information

**A surgery-informed precision approach to determining brain targets for real-time fMRI**

**neurofeedback modulation in chronic pain**

Dan Liu ^a,b^ , Yiqi Mi^b^ , Menghan Li ^a,b^ , Anna Nigri ^c^ , Marina Grisoli ^c^, Keith M Kendrick ^a,b^ , Benjamin Becker ^d,e^, Stefania Ferraro ^a,b,*^

**Affiliations**

^a^ The Center of Psychosomatic Medicine, Sichuan Provincial Center for Mental Health, Sichuan Provincial People’s Hospital University of Electronic Science and Technology of China, Chengdu, China

^b^ School of Life Science and Technology, University of Electronic Science and Technology of China, Chengdu, China

^c^ Neuroradiology Department, Neurological Institute Carlo Besta, Milan, Italy

^d^ State Key Laboratory of Brain and Cognitive Sciences, The University of Hong Kong, Hong Kong, China

^e^ Department of Psychology, The University of Hong Kong, Hong Kong, China

**Methods**

**rt-fMRI-NF records selection**

Records were screened by two independent reviewers (LD and YM) based on the following criteria: (1) written in English and peer-reviewed; (2) reporting an original study (excluding reviews or commentaries and not duplicated results); (3) using only rt-fMRI-NF training paradigm to modulate the level of pain in healthy participants or chronic pain patients. For the included records, the first reviewer (LD) extracted: (1) demographic information (i.e., population, sample size, and age and gender distribution); (2) feedback characteristics (method of localization of the target and type of feedback); (3) suggested conditioning strategy and direction of request for modulation of target activity (increased or decreased); (4) experimental design (i.e., number of training sessions, use of a control group or control condition); (5) coordinates of the brain targets in the standardized space [Talairach & Tournoux (TAL) or Montreal Neurological Institute (MNI)]; (6) behavioral effects on pain level. The second reviewer (YM) independently validated the extracted data: when discrepancies were observed, they were jointly discussed with the senior author of the paper (SF) to reach a final consensus. The original coordinates expressed in the TAL space were converted to MNI stereotactic space using GingerALE (3.0.2). In consideration that several nomenclatures and subdivisions exist for brain areas, and in particular for the cingulate cortex (Vogt, 2009), we decided to identify the target regions uniquely, thus independently from the nomenclature given by the Authors, relying on the Automated Anatomical Labelling Atlas 3 (AAL3) (Rolls et al., 2020) and the probabilistic atlas of the insula cortex (Faillenot et al., 2017).

**Functional Surgery Records Selection**

Among the identified papers (from the already published meta-analyses and the more recent studies), records were then selected based on the following criteria: published after 2000, treating at least 8 patients, with a clear description of the target landmarks, resulting in pain relief in at least 40% of patients within the longest follow-up. Since our interest was in defining the location of the target, where authors of a study localized the target based on a previous study, we utilized the study that reported more detailed information about its location. In addition, when the technique was developed and refined in the same region by the same group, assuming that the latest development was a better target than the previous one, as in the case of thalamotomy with MRgFUS (Gallay et al., 2023), we used the description of the last published work. Moreover, if a paper reported multiple targets in identical regions, we computed their center of mass (Gallay et al., 2023; Strauss et al., 2018). For each included record, the first reviewer (LD) extracted (1) demographic information, (2) type of chronic pain, (3) behavioral effects on pain level, and (4) information relevant to identifying the coordinates of the target. The second reviewer (YM) independently validated the extracted data. When discrepancies were observed, they were jointly discussed with the senior author of the paper (SF) to reach a final consensus. Also in this case, we decided to identify the cortical target regions uniquely, thus independently from the nomenclature given by the Authors, relying on AAL3 atlas (Rolls et al., 2020). Based on the collected information relative to the localization of the target, three senior researchers (SF, AN, and LM in the acknowledgment note) computed independently the coordinates of each target in the MNI space. with MANGO v4.1 (http://rii.uthscsa.edu/mango/) employing the MNI template ICBM 2009b Nonlinear Symmetric template [spatial resolution: 0.5x0.5x0.5mm; mni icbm152 t1 tal nlin sym 09b hires. nii, ICBM 152 Nonlinear atlases (2009) – NIST (mcgill.ca)]. Then, for each target, after verifying that the distance between the points located by the first reviewer relative to the second and third reviewers was not greater than the arbitrary distance of 5 mm, the center of mass of the located points was calculated. As the last step, due to the complexity of the thalamic anatomy and the inherent difficulties in defining the coordinates based on the text of the selected studies, we verified the localization of the extracted coordinates on the in-vivo high-resolution structural-MRI human thalamic atlas (Saranathan et al., 2021). If the reported coordinates were not consistent with the location of the thalamic nuclei of interest, we first wrote to the Authors of the paper to ask for clarifications and, in case of no responses within one month, we used the coordinates of the centroid of the putative thalamic nucleus computed on the same atlas.

***Identification of the rs-fMRI networks underlying the target regions***

To identify the underlying rs-fMRI networks, Marsbar (v 0.44) was used to create 6 mm (for cortical targets) and 3 mm (for subcortical targets) radius regions of interest (ROIs) centered in the identified MNI coordinates. The 3 mm radius for the subcortical ROIs was chosen because it avoided the overlap between the ROIs from different but nearby regions. To clarify, the targets for rt-fMRI-NF were always on either the left or right side of specific structures, while the targets for funcSurg were always bilateral. Therefore, when computing the functional connectivity for rt-fMRI-NF ROIs, we employed the single lateralized ROI as a seed. In contrast, for funcSurg ROIs, we employed the left and right ROIs together as a seed. Using CONN toolbox v22a ([www.nitrc.org/projects/conn](http://www.nitrc.org/projects/conn)) (Nieto-Castanon, 2020), we calculated the seed-based functional connectivity of each ROI (as seed for rt-fMRI-NF) or a couple of ROIs (for funcSurg) employing rs-fMRI data from 30 subjects (age: M = 29.17ys, SD = 3.32ys; 20 females) of the Young Adult HCP dataset (Smith et al., 2013; Van Essen et al., 2013), for details, see <https://www.humanconnectome.org/hcp-protocols-ya-7t-imaging>). As declared by HCP, all participants provided written informed consent to the study and the sharing of de-identified data. For each subject, we employed the four rs-fMRI data runs acquired at 7T (900 volumes per run, 1.6 mm isotropic voxels, TR = 1000 ms, TE = 22.2 ms, flip angle = 45 degrees, FOV = 208 × 208 mm; <https://www.humanconnectome.org/hcp-protocols-ya-7t-imaging>) (Smith et al., 2013; Uğurbil et al., 2013) and which have been already preprocessed (HCP filename: ‘rfMRI hp2000 clean.nii.gz’). The HCP preprocessing steps comprised gradient nonlinearity-induced distortion correction, rigid body head motion correction, EPI image distortion correction, co-registration between the fMRI and structural data, normalization to MNI space, high-pass filtering, brain masking (Glasser et al., 2013), and independent components analysis based artifact removal of noise components (Salimi-Khorshidi et al., 2014). As structural MRI data, we used 3T T1-weighted images re-sampled at 1.6 mm resolution provided by the HCP for the use with 7T rs-fMRI dataset (filename: ‘T1w restore.1.6.nii.gz’). Using CONN toolbox v21a (www.nitrc.org/projects/conn), the preprocessed fMRI data from HCP underwent the following preprocessing and denoising steps: segmentation, artifact identification using Artifact Detection Tools (ART), smoothing (FWHM = 6 mm), and standard denoising pipeline (aCompCor). For each ROI or a couple of ROIs, seed-based connectivity (SBC) maps were estimated as Fisher-transformed bivariate correlation coefficients (weighted-GLM) (Nieto-Castanon, 2020). Multivariate parametric statistics with random effects across subjects and sample covariance estimation across multiple measurements were applied (cluster threshold: p < 0.05 cluster-level, p-FDR corrected; voxel threshold: p < 0.001 uncorrected).

***Principal components analysis of the identified rs-fMRI networks (PCA rs-fMRI-derived maps)***

Separately for the rt-fMRI-NF and the funcSurg targets, we performed a dimensionality reduction with principal components analysis (PCA) of the obtained rs-fMRI maps (8 for rt-fMRI-NF and 8 for funcSurg). This step was crucial not only for reducing the complexity of our data but also for identifying the most significant patterns within the functional connectivity networks. To achieve this, we utilized the PCA class from the *scikit-learn* library in Python (version 3.11.7). For both analyses, we retained only the first components that explain a cumulative variance of at least 80%. Then, we obtained the maps of the rs-fMRI data that reflect the information captured by the principal components (from now on, these maps are named PCA rs-fMRI-derived maps). Next, we asked whether the PCA rs-fMRI-derived maps significantly overlapped with the salience, sensorimotor, and default mode (DMN) networks (herein after named canonical rs-fMRI networks). To this aim, we used a null map approach (Burt et al., 2020) as described in Ferraro et al.(Ferraro et al., 2022), employing the rs-fMRI network maps available at <http://findlab.stanford.edu/research.html> (Shirer et al., 2012). Briefly, we generated 1000 surrogate maps for each canonical rs-fMRI map (i.e., salience, DMN, and sensorimotor). Then, we computed the null distribution of the overlap scores, computing the number of overlapping voxels between the PCA rs-fMRI-derived map of interest and each of the surrogate maps. Using this null distribution, we then obtained a p-value testing the hypothesis that the overlap between the PCA rs-fMRI-derived map and the canonical rs-fMRI map was greater than expected by chance. To preserve spatial autocorrelation (Markello et al., 2021), the surrogate maps were generated employing the algorithm implemented in Neuromaps (Markello et al., 2022) and described in Burt et al. (Burt et al., 2020).

***Neurotransmitter receptors profiling of PCA maps***

Overall, the following neurotransmitter and neuromodulatory systems were investigated: gamma-aminobutyric acid type A (GABA) (Dukart et al. 2018) serotonin 5-hydroxytryptamine receptor subtype 1a (5-HT1a) (Hansen et al., 2022; Savli et al., 2012) and 1b (Beliveau et al., 2017; Savli et al., 2012) serotonin transporter (SERT) (Beliveau et al., 2017; Savli et al., 2012), dopamine D2 (D2) (Alakurtti et al., 2015), dopamine transporter (DAT) (Dukart et al., 2018), noradrenaline transporter (NAT) (Hesse et al., 2017), and opioid (MU and KappaOp) (Hansen et al., 2022; Kantonen et al., 2020; Shokri-Kojori et al., 2022). Using JuSpace, the PCA rs-fMRI-derived maps, and the neurotransmitter receptor maps were parcellated into 116 regions according to the Automated Anatomical Labeling (AAL) atlas, from which mean regional values were extracted. Adjusting for spatial autocorrelation through partial correlation with grey matter probability estimates; Spearman’s correlation (Fisher’s z-transformed value) was computed between each PCA rs-fMRI-derived map and each neurotransmitter receptor map. Then, the exact p-value of each correlation was calculated by generating randomly permuted neurotransmitter receptor maps (n=1000) that maintain the same spatial autocorrelation as the original map. These permuted maps were then used to create a null distribution, against which each PCA rs-fMRI-derived map is compared to determine if the observed correlation was significant (p-value < 0.0017, Bonferroni correction for 29 statistical tests to control for multiple comparisons).

**RESULTS**

***Description of the selected rt-fMRI-NF studies***

DeCharms et al. (DeCharms et al., 2005) used rt-fMRI-NF to study how the willful modulation of MCC affects acute pain and chronic pain. Testing 8 healthy participants and 4 control groups under different conditions, they observed that only the experimental group learned to modulate the activity of the MCC and concomitantly reported a reduction in the level of perceived acute pain. In the same study, they investigated 12 subjects (8 experimental and 4 control) with chronic pain observing that the regulation of MCC activity reduced the level of perceived chronic pain. However, a subsequent study by the same group on a large sample did not replicate these results (Guan et al., 2015). Emmert et al. (Emmert et al., 2014) studied in two groups of healthy subjects (7 subjects per group) whether the willful modulation of subgenual ACC and antIns activity decreased the level of acute pain perception. Both groups reduced pain during the rt-fMRI-NF, but the Authors reported no difference in the level of pain between regulators (participants able to change the activity of the selected target) and non-regulators. Rance et al.(Rance et al., 2014b) showed that 10 healthy subjects learned to downregulate left pIns and subgenual ACC and upregulate left pIns consistently but not subgenual ACC. Despite the learned ability, successful regulation was not linked to a reduction of pain intensity. Similarly, in their second study (Rance et al., 2014a), they trained participants to increase the difference in subgenual ACC and left pIns activity, but again, they did not find significant changes in pain perception. Guan et al. (Guan et al., 2015), in a double-blinded randomized study, showed that 6 out of 8 post-herpetic neuralgia patients learned to regulate pregenual ACC activity with significant pain reduction.

**References**

Alakurtti, K., Johansson, J. J., Joutsa, J., Laine, M., Bäckman, L., Nyberg, L., & Rinne, J. O. (2015). Long-term test-retest reliability of striatal and extrastriatal dopamine D2/3 receptor binding: Study with [(11)C]raclopride and high-resolution PET. *Journal of Cerebral Blood Flow and Metabolism: Official Journal of the International Society of Cerebral Blood Flow and Metabolism*, *35*(7), 1199–1205. https://doi.org/10.1038/jcbfm.2015.53

Beliveau, V., Ganz, M., Feng, L., Ozenne, B., Højgaard, L., Fisher, P. M., Svarer, C., Greve, D. N., & Knudsen, G. M. (2017). A High-Resolution In Vivo Atlas of the Human Brain’s Serotonin System. *The Journal of Neuroscience: The Official Journal of the Society for Neuroscience*, *37*(1), 120–128. https://doi.org/10.1523/JNEUROSCI.2830-16.2016

Burt, J. B., Helmer, M., Shinn, M., Anticevic, A., & Murray, J. D. (2020). Generative modeling of brain maps with spatial autocorrelation. *NeuroImage*, *220*, 117038. https://doi.org/10.1016/j.neuroimage.2020.117038

deCharms, R. C., Maeda, F., Glover, G. H., Ludlow, D., Pauly, J. M., Soneji, D., Gabrieli, J. D. E., & Mackey, S. C. (2005). Control over brain activation and pain learned by using real-time functional MRI. *Proceedings of the National Academy of Sciences*, *102*(51), 18626–18631. https://doi.org/10.1073/pnas.0505210102

Dukart, J., Holiga, Š., Chatham, C., Hawkins, P., Forsyth, A., McMillan, R., Myers, J., Lingford-Hughes, A. R., Nutt, D. J., Merlo-Pich, E., Risterucci, C., Boak, L., Umbricht, D., Schobel, S., Liu, T., Mehta, M. A., Zelaya, F. O., Williams, S. C., Brown, G., … Sambataro, F. (2018). Cerebral blood flow predicts differential neurotransmitter activity. *Scientific Reports*, *8*(1), 4074. https://doi.org/10.1038/s41598-018-22444-0

Emmert, K., Breimhorst, M., Bauermann, T., Birklein, F., Van De Ville, D., & Haller, S. (2014). Comparison of anterior cingulate vs. Insular cortex as targets for real-time fMRI regulation during pain stimulation. *Frontiers in Behavioral Neuroscience*, *8*, 350. https://doi.org/10.3389/fnbeh.2014.00350

Faillenot, I., Heckemann, R. A., Frot, M., & Hammers, A. (2017). Macroanatomy and 3D probabilistic atlas of the human insula. *NeuroImage*, *150*, 88–98. https://doi.org/10.1016/j.neuroimage.2017.01.073

Ferraro, S., Klugah-Brown, B., Tench, C. R., Bazinet, V., Bore, M. C., Nigri, A., Demichelis, G., Bruzzone, M. G., Palermo, S., Zhao, W., Yao, S., Jiang, X., Kendrick, K. M., & Becker, B. (2022). The central autonomic system revisited—Convergent evidence for a regulatory role of the insular and midcingulate cortex from neuroimaging meta-analyses. *Neuroscience and Biobehavioral Reviews*, *142*, 104915. https://doi.org/10.1016/j.neubiorev.2022.104915

Gallay, M. N., Magara, A. E., Moser, D., Kowalski, M., Kaeser, M., & Jeanmonod, D. (2023). Magnetic resonance-guided focused ultrasound central lateral thalamotomy against chronic and therapy-resistant neuropathic pain: Retrospective long-term follow-up analysis of 63 interventions. *Journal of Neurosurgery*, 1–10. https://doi.org/10.3171/2023.1.JNS222879

Glasser, M. F., Sotiropoulos, S. N., Wilson, J. A., Coalson, T. S., Fischl, B., Andersson, J. L., Xu, J., Jbabdi, S., Webster, M., Polimeni, J. R., Van Essen, D. C., Jenkinson, M., & WU-Minn HCP Consortium. (2013). The minimal preprocessing pipelines for the Human Connectome Project. *NeuroImage*, *80*, 105–124. https://doi.org/10.1016/j.neuroimage.2013.04.127

Guan, M., Ma, L., Li, L., Yan, B., Zhao, L., Tong, L., Dou, S., Xia, L., Wang, M., & Shi, D. (2015). Self-Regulation of Brain Activity in Patients with Postherpetic Neuralgia: A Double-Blind Randomized Study Using Real-Time fMRI Neurofeedback. *PLOS ONE*, *10*(4), e0123675. https://doi.org/10.1371/journal.pone.0123675

Hansen, J. Y., Shafiei, G., Markello, R. D., Smart, K., Cox, S. M. L., Nørgaard, M., Beliveau, V., Wu, Y., Gallezot, J.-D., Aumont, É., Servaes, S., Scala, S. G., DuBois, J. M., Wainstein, G., Bezgin, G., Funck, T., Schmitz, T. W., Spreng, R. N., Galovic, M., … Misic, B. (2022). Mapping neurotransmitter systems to the structural and functional organization of the human neocortex. *Nature Neuroscience*, *25*(11), 1569–1581. https://doi.org/10.1038/s41593-022-01186-3

Hesse, S., Becker, G.-A., Rullmann, M., Bresch, A., Luthardt, J., Hankir, M. K., Zientek, F., Reißig, G., Patt, M., Arelin, K., Lobsien, D., Müller, U., Baldofski, S., Meyer, P. M., Blüher, M., Fasshauer, M., Fenske, W. K., Stumvoll, M., Hilbert, A., … Sabri, O. (2017). Central noradrenaline transporter availability in highly obese, non-depressed individuals. *European Journal of Nuclear Medicine and Molecular Imaging*, *44*(6), 1056–1064. https://doi.org/10.1007/s00259-016-3590-3

Kantonen, T., Karjalainen, T., Isojärvi, J., Nuutila, P., Tuisku, J., Rinne, J., Hietala, J., Kaasinen, V., Kalliokoski, K., Scheinin, H., Hirvonen, J., Vehtari, A., & Nummenmaa, L. (2020). Interindividual variability and lateralization of μ-opioid receptors in the human brain. *NeuroImage*, *217*, 116922. https://doi.org/10.1016/j.neuroimage.2020.116922

Markello, R. D., Arnatkeviciute, A., Poline, J.-B., Fulcher, B. D., Fornito, A., & Misic, B. (2021). Standardizing workflows in imaging transcriptomics with the abagen toolbox. *eLife*, *10*, e72129. https://doi.org/10.7554/eLife.72129

Markello, R. D., Hansen, J. Y., Liu, Z.-Q., Bazinet, V., Shafiei, G., Suárez, L. E., Blostein, N., Seidlitz, J., Baillet, S., Satterthwaite, T. D., Chakravarty, M. M., Raznahan, A., & Misic, B. (2022). neuromaps: Structural and functional interpretation of brain maps. *Nature Methods*, *19*(11), 1472–1479. https://doi.org/10.1038/s41592-022-01625-w

Nieto-Castanon, A. (2020). *Handbook of functional connectivity magnetic resonance imaging methods in CONN*. https://doi.org/10.56441/hilbertpress.2207.6598

Rance, M., Ruttorf, M., Nees, F., Schad, L. R., & Flor, H. (2014a). Neurofeedback of the difference in activation of the anterior cingulate cortex and posterior insular cortex: Two functionally connected areas in the processing of pain. *Frontiers in Behavioral Neuroscience*, *8*, 357. https://doi.org/10.3389/fnbeh.2014.00357

Rance, M., Ruttorf, M., Nees, F., Schad, L. R., & Flor, H. (2014b). Real time fMRI feedback of the anterior cingulate and posterior insular cortex in the processing of pain: Real Time fMRI Feedback During Pain. *Human Brain Mapping*, *35*(12), 5784–5798. https://doi.org/10.1002/hbm.22585

Rolls, E. T., Huang, C.-C., Lin, C.-P., Feng, J., & Joliot, M. (2020). Automated anatomical labelling atlas 3. *NeuroImage*, *206*, 116189. https://doi.org/10.1016/j.neuroimage.2019.116189

Salimi-Khorshidi, G., Douaud, G., Beckmann, C. F., Glasser, M. F., Griffanti, L., & Smith, S. M. (2014). Automatic denoising of functional MRI data: Combining independent component analysis and hierarchical fusion of classifiers. *NeuroImage*, *90*, 449–468. https://doi.org/10.1016/j.neuroimage.2013.11.046

Saranathan, M., Iglehart, C., Monti, M., Tourdias, T., & Rutt, B. (2021). In vivo high-resolution structural MRI-based atlas of human thalamic nuclei. *Scientific Data*, *8*, 275. https://doi.org/10.1038/s41597-021-01062-y

Savli, M., Bauer, A., Mitterhauser, M., Ding, Y.-S., Hahn, A., Kroll, T., Neumeister, A., Haeusler, D., Ungersboeck, J., Henry, S., Isfahani, S. A., Rattay, F., Wadsak, W., Kasper, S., & Lanzenberger, R. (2012). Normative database of the serotonergic system in healthy subjects using multi-tracer PET. *NeuroImage*, *63*(1), 447–459. https://doi.org/10.1016/j.neuroimage.2012.07.001

Shirer, W. R., Ryali, S., Rykhlevskaia, E., Menon, V., & Greicius, M. D. (2012). Decoding subject-driven cognitive states with whole-brain connectivity patterns. *Cerebral Cortex (New York, N.Y.: 1991)*, *22*(1), 158–165. https://doi.org/10.1093/cercor/bhr099

Shokri-Kojori, E., Naganawa, M., Ramchandani, V. A., Wong, D. F., Wang, G.-J., & Volkow, N. D. (2022). Brain opioid segments and striatal patterns of dopamine release induced by naloxone and morphine. *Human Brain Mapping*, *43*(4), 1419–1430. https://doi.org/10.1002/hbm.25733

Smith, S. M., Beckmann, C. F., Andersson, J., Auerbach, E. J., Bijsterbosch, J., Douaud, G., Duff, E., Feinberg, D. A., Griffanti, L., Harms, M. P., Kelly, M., Laumann, T., Miller, K. L., Moeller, S., Petersen, S., Power, J., Salimi-Khorshidi, G., Snyder, A. Z., Vu, A. T., … WU-Minn HCP Consortium. (2013). Resting-state fMRI in the Human Connectome Project. *NeuroImage*, *80*, 144–168. https://doi.org/10.1016/j.neuroimage.2013.05.039

Strauss, I., Berger, A., Ben Moshe, S., Arad, M., Hochberg, U., Gonen, T., & Tellem, R. (2018). Double Anterior Stereotactic Cingulotomy for Intractable Oncological Pain. *Stereotactic and Functional Neurosurgery*, *95*(6), 400–408. https://doi.org/10.1159/000484613

Uğurbil, K., Xu, J., Auerbach, E. J., Moeller, S., Vu, A. T., Duarte-Carvajalino, J. M., Lenglet, C., Wu, X., Schmitter, S., Van de Moortele, P. F., Strupp, J., Sapiro, G., De Martino, F., Wang, D., Harel, N., Garwood, M., Chen, L., Feinberg, D. A., Smith, S. M., … Yacoub, E. (2013). Pushing spatial and temporal resolution for functional and diffusion MRI in the Human Connectome Project. *NeuroImage*, *80*, 80–104. https://doi.org/10.1016/j.neuroimage.2013.05.012

Van Essen, D. C., Smith, S. M., Barch, D. M., Behrens, T. E. J., Yacoub, E., & Ugurbil, K. (2013). The WU-Minn Human Connectome Project: An overview. *NeuroImage*, *80*, 62–79. https://doi.org/10.1016/j.neuroimage.2013.05.041

Vogt, B. A. (2009). Regions and Subregions of the Cingulate Cortex. In B. A. Vogt (Ed.), *Cingulate Neurobiology and Disease* (p. 0). Oxford University Press. https://doi.org/10.1093/oso/9780198566960.003.0001

| **Table S1** | | | | | | | | |
| --- | --- | --- | --- | --- | --- | --- | --- | --- |
| **Author** | **Target (as reported)** | **Condition** | **Sample** | **Population** | **Regulation** | **Strategy** | **Noxious Stimulation** | **Behavioural effects on pain** |
| DeCharms et al. (2005) | rACC | E:NF | 12 | Patients with neuropathic pain or fibromyalgia (#1) | Up/down | Attentional changes or reappraisal of the stimuli | / | YES |
|  |  | C: autonomic biofeedback |  |  |  |  |  |  |
|  |  | E:NF | 36 | Healthy |  | Control of pain perception | Thermal |  |
|  |  | C:No or SHAM feedback |  |  |  |  |  |  |
| Emmert et al. (2014) | L aIns | E:NF | 14 | Healthy | Down | Free | Thermal | NO |
|  | ACC |  | 14 |  |  |  |  |  |
| Rance et al. (2014a) | ACC | E:NF | 10 | Healthy | Up/down | Free | Electrical | NO |
|  | L pIns |  |  |  |  |  |  |  |
| Rance et al. (2014b) | ACC | E:NF | 10 | Healthy | Up/down | Free | Electrical | NO |
|  | L pIns |  |  |  |  |  |  |  |
| Guan et al. (2015) | rACC | E:NF | 8 | Patients with neuropathic pain (PHN) | Up/down | Attentional changes or reappraisal of the stimuli | Mechanical | YES |
|  | PCC | C:NF | 8 |  |  |  |  |  |
| **Table S1** Selected rt-fMRI-NF studies.Abbreviations: rt-fMRI-NF, real-time fMRI neurofeedback; E, experimental group; C, control group; NF, neurofeedback; ACC, anterior cingulate cortex; PCC, posteriorcingulate cortex; pIns, posterior insula; alns, anterior insula; PHN, post-herpetic neuralgia | | | | | | | | |

| **Table S2** | | | | | | |
| --- | --- | --- | --- | --- | --- | --- |
| **Author** | **Target** | **Target regions identified** | **Population** | **Sample** | **Technique** | **Stimulation effects on pain** |
| Rasche et al. (2006) | VPL/VPM | “The coordinate for the lateral somatosensory thalamus was as follows: Y = 3 to 5 mm anterior to the posterior commissure; Z = 0 to –2 mm below the inter commissural line; and X = 10 to 12 mm lateral to the midline for facial pain, 12 to 15 mm for pain in the upper extremity, and 15 to 18 mm for pain in the lower extremity.” | Failed back surgery syndrome, neuropathic pain of peripheral origin, dysesthesia dolorosa, phantom-limb pain, central pain syndromes, poststroke pain, postherpetic neuralgia, nociceptive pain | 56 | DBS of thalamic nuclei | “The best long-term results were seen in patients with failed–Back Surgery Syndrome. Patients with peripheral neuropathic pain and Dysesthesia Dolorosa responded very well to DBS. The results in Spinal Cord Injury patients with central pain syndromes were less favorable. Disappointing outcomes for patients with thalamic pain syndrome or post-stroke pain.” |
| Boccard et al. (2017) | MCC | “The target used was 20mm posterior to the anterior tip of the frontal horns of the lateral ventricles.” | Failed back surgery syndrome, post-stroke pain, brachial plexus injury, spinal cord injury, head injury, road traffic accident, pain of unknown cause | 24 | DBS in cingulate cortex | “The NRS dropped significantly.” |
| Abreu et al. (2017) | VPL/VPM | “VPL found 10–13 mm lateral to the posterior commissure.” | Phantom limb pain, deafferentation pain | 16 | DBS of thalamic nuclei | “Patient-reported outcome measure improvements were clear after three years in this cohort. The VAS and UWNPS , and BPI scores have improved with statistical significance after one month. Throughout the third year, mean pain relief was sustained.” |
| Abdallat et al. (2021) | CM/pf VPL/VPM | “The CM–Pf targets were x = 8 mm lateral to the inter-commissural line, y = 8 mm posterior to the mid-commissural point, and z = 0 at the level of the inter-commissural plane. Target coordinates for the VPL were x = 14, y = -10, and z = 0.” | Facial pain, complex regional pain syndrome, poststroke/central pain, central thalamic pain, postherpetic pain, deafferentation pain, brachial plexus injury, failed back surgery syndrome with neuropathic pain, neuropathic pain, and phantom limb pain | 40 | DBS of thalamic nuclei | “Out of the total group of 40 patients, 33 (82.5%) indicated that they had a clear benefit during test stimulation.” |
| Strauss et al. (2018) | MCC | “The target on each side, 24 mm behind the tip of the frontal horn, 7–8 mm from the midline the second lesion was performed more anteriorly, 16 mm behind the tip of the frontal horn.” | Medically refractory oncological pain | 13 | Surgical cingulotomy | “All patients reported significant pain relief immediately after the operation in the recovery room (mean VAS 0.9 ± 1.3). Eight of the 11 patients (72.7%) reported significant pain relief (>50%), and 1 patient (9%) reported partial improvement. In 2 patients, the pain returned to its original severity.” |
| Yen et al. (2005) | MCC | “The target slice was selected as 24 mm posterior to the anterior tip of the lateral ventricle.” | Medically intractable pain | 22 | Surgical cingulotomy | “Of the 15 patients with cancer pain, significant or meaningful pain relief was achieved in 67% of patients at one-month follow-up, which decreased to 58% at three months and 50% at six months. Of the seven patients with intractable pain from non-neoplastic origin, four achieved significant pain relief, one obtained meaningful relief, and two reported no change at one year follow-up.” |
| Gallay et al. (2023) | CLp | “This corresponds to the anteroposterior position of the CLp, centered between 3 mm anterior and 1 mm posterior to the posterior commissure.” | Chronic and therapy-resistant neuropathic pain | 55 | MRgFUS thalamotomy | “The mean pain relief rated by patients was 42% ± 32% at 3 months, 43% ± 36% at 1 year, and 42% ± 37% at the last follow-up (n = 63).” |
| Urgosik et al. (2018) | CM/pf | “The CM/Pf complex was localized 4–6 mm lateral to the wall of the third ventricle, 8 mm posterior to the midpoint, and 2–3 mm superior to the inter-commissural line.” | Trigeminal neuralgia, trigeminal neuropathic pain, thalamic pain, phantom pain, causalgic pain, and facial pain | 30 | Gamma knife thalamotomy | “Initial successful results were achieved in 13 (43.3%) of the patients, with complete pain relief in 1 of these patients.” |
| Lovo et al. (2019) | CM/pf | “The best coordinates of CM-pf were X: 5.5 mm from the thalamic border, Y: 3.7 mm anterior to the posterior commissure, and Z: 3.7 mm from the inter-commissural line.” | Refractory trigeminal pain  other complex pain syndromes | 14 | Gamma knife thalamotomy | “90% of patients reported some form of relief, the average VAS at the time of response was 3.5(range: 0-9), and the average time to response was 67.3 days (range: 2-210). The neuromodulation effect of radiation was seen in 60% of patients.” |
| **Table S2** Selected funcSurg studies. Abbreviations: MCC, middle cingulate cortex; CLp, central lateral thalamus; CM/Pf, centromedian/parafascicular nucleus; VPL, ventral posterior lateral nucleus; VPM, ventral posterior medial nucleus; DBS, Deep Brain Stimulation; VAS, Visual Analog Scale; NRS, Numerical Rating Scale. | | | | | | |

Table S3

|  | **AAL Region** | **# Voxels** | **Peak coordinate [x,y,z]** |
| --- | --- | --- | --- |
|  |  |  |  |
| **rt-fMRI-NF PCA comp1** | Cingulum_Ant_L | 800 | [0, 46, 2] |
|  | Cingulum_Ant_R | 516 | [10, 46, 2] |
|  | Frontal_Sup_Medial_L | 237 | [-4, 56, 4] |
|  | Cingulum_Mid_R | 232 | [0, -18, 30] |
|  | Precuneus_L | 192 | [-10, -72, 36] |
|  | Precuneus_R | 176 | [12, -68, 38] |
|  | Frontal_Sup_Medial_R | 141 | [10, 46, 0] |
|  | Cingulum_Mid_L | 138 | [0, -18, 32] |
|  | Frontal_Med_Orb_R | 132 | [10, 42, -4] |
|  | Cingulum_Post_L | 124 | [0, -34, 28] |
|  | Frontal_Med_Orb_L | 72 | [-8, 54, -2] |
|  | Insula_L | 70 | [-36, 14, -12] |
|  | Insula_R | 68 | [30, 16, -12] |
|  | Cingulum_Post_R | 66 | [4, -40, 26] |
|  | Cuneus_L | 55 | [-10, -72, 30] |
| **rt-fMRI-NF PCA comp2** | Postcentral_L | 1923 | [-46, -12, 50] |
|  | Postcentral_R | 1030 | [68, -16, 22] |
|  | Temporal_Sup_L | 879 | [-42, -22, 2] |
|  | Rolandic_Oper_R | 647 | [68, -10, 12] |
|  | Precentral_R | 644 | [66, 4, 20] |
|  | Rolandic_Oper_L | 608 | [-38, -18, 18] |
|  | Temporal_Sup_R | 575 | [70, -32, 12] |
|  | Insula_R | 526 | [50, -4, 2] |
|  | Precentral_L | 501 | [-14, -12, 68] |
|  | Insula_L | 364 | [-36, -12, 14] |
|  | Cingulum_Ant_R | 333 | [12, 40, 10] |
|  | Supp_Motor_Area_L | 307 | [0, -2, 56] |
|  | Cingulum_Mid_L | 304 | [0, -28, 50] |
|  | Cingulum_Ant_L | 301 | [-4, 46, 14] |
|  | Cingulum_Mid_R | 283 | [2, 8, 44] |
|  | Supp_Motor_Area_R | 262 | [4, -4, 60] |
|  | SupraMarginal_R | 229 | [68, -26, 18] |
|  | Precuneus_L | 227 | [0, -40, 58] |
|  | SupraMarginal_L | 218 | [-64, -24, 16] |
|  | Occipital_Mid_L | 204 | [-28, -86, 38] |
|  | Parietal_Inf_L | 186 | [-42, -48, 50] |
|  | Parietal_Sup_L | 184 | [-18, -50, 66] |
|  | Heschl_L | 178 | [-38, -22, 12] |
|  | Precuneus_R | 150 | [12, -48, 70] |
|  | Paracentral_Lobule_L | 144 | [0, -38, 54] |
|  | Frontal_Sup_Medial_L | 138 | [-4, 16, 42] |
|  | Heschl_R | 130 | [58, -10, 8] |
|  | Frontal_Sup_Medial_R | 120 | [2, 58, 14] |
|  | Frontal_Med_Orb_R | 99 | [8, 40, -8] |
|  | Cerebelum_6_R | 95 | [28, -76, -24] |
|  | Cerebelum_Crus1_R | 80 | [10, -84, -24] |
|  | Temporal_Inf_L | 70 | [-42, -62, -6] |
|  | Cerebelum_8_R | 66 | [26, -54, -50] |
|  | Paracentral_Lobule_R | 65 | [12, -38, 54] |
|  | Temporal_Mid_L | 64 | [-52, -64, -4] |
|  | Angular_L | 59 | [-44, -78, 30] |
|  | Cingulum_Post_L | 56 | [0, -36, 28] |
|  | Frontal_Mid_L | 55 | [-24, 12, 62] |
|  | Cerebelum_4_5_R | 54 | [24, -40, -24] |
| **funcSurg PCA comp1** | Insula_R | 974 | [34, 24, 6] |
|  | Postcentral_L | 932 | [-20, -28, 64] |
|  | Precentral_R | 894 | [22, -30, 66] |
|  | Postcentral_R | 847 | [58, -20, 30] |
|  | Rolandic_Oper_R | 812 | [58, -20, 18] |
|  | Insula_L | 803 | [-34, 24, 6] |
|  | Supp_Motor_Area_R | 795 | [12, 14, 70] |
|  | Supp_Motor_Area_L | 762 | [0, 12, 44] |
|  | Precentral_L | 720 | [-20, -24, 74] |
|  | Rolandic_Oper_L | 716 | [-48, -12, 18] |
|  | Cingulum_Mid_R | 708 | [12, 12, 38] |
|  | SupraMarginal_R | 566 | [58, -22, 26] |
|  | Cingulum_Mid_L | 541 | [0, 12, 34] |
|  | SupraMarginal_L | 533 | [-54, -32, 34] |
|  | Paracentral_Lobule_R | 434 | [12, -26, 58] |
|  | Frontal_Sup_R | 369 | [30, 10, 66] |
|  | Cingulum_Ant_L | 346 | [2, 28, 28] |
|  | Paracentral_Lobule_L | 342 | [-10, -34, 66] |
|  | Calcarine_L | 325 | [-6, -68, 14] |
|  | Frontal_Inf_Oper_L | 311 | [-62, 8, 8] |
|  | Frontal_Mid_R | 301 | [34, 50, 20] |
|  | Precuneus_R | 298 | [14, -56, 18] |
|  | Frontal_Mid_L | 271 | [-30, 8, 58] |
|  | Precuneus_L | 258 | [-10, -54, 12] |
|  | Frontal_Inf_Oper_R | 223 | [40, 10, 8] |
|  | Calcarine_R | 209 | [18, -60, 14] |
|  | Frontal_Sup_Medial_L | 204 | [0, 18, 40] |
|  | Cuneus_L | 204 | [-8, -70, 26] |
|  | Temporal_Sup_L | 198 | [-42, 2, -10] |
|  | Cingulum_Ant_R | 192 | [2, 26, 24] |
|  | Parietal_Inf_L | 152 | [-54, -32, 36] |
|  | Frontal_Sup_L | 151 | [-16, 6, 64] |
|  | Cuneus_R | 144 | [8, -82, 16] |
|  | Temporal_Sup_R | 136 | [54, 0, 2] |
|  | Frontal_Inf_Tri_L | 125 | [-32, 32, 2] |
|  | Temporal_Pole_Sup_L | 94 | [-52, 4, 0] |
|  | Heschl_R | 80 | [54, -10, 8] |
|  | Temporal_Pole_Sup_R | 61 | [56, 10, -4] |
|  | Putamen_R | 57 | [32, 12, 4] |
|  | Heschl_L | 54 | [-40, -20, 2] |
|  | Fusiform_L | 51 | [-28, -62, -12] |
|  | Cerebelum_8_R | 51 | [30, -48, -44] |
| **funcSurg PCA comp2** | Cingulum_Mid_L | 423 | [-2, 6, 38] |
|  | SupraMarginal_L | 406 | [-62, -22, 36] |
|  | Cingulum_Mid_R | 384 | [8, -34, 46] |
|  | Precuneus_R | 341 | [8, -54, 68] |
|  | SupraMarginal_R | 326 | [60, -26, 34] |
|  | Insula_R | 296 | [42, -4, 8] |
|  | Postcentral_R | 277 | [62, -18, 30] |
|  | Temporal_Sup_L | 252 | [-40, -16, -4] |
|  | Precuneus_L | 250 | [-8, -48, 64] |
|  | Calcarine_R | 228 | [16, -58, 12] |
|  | Calcarine_L | 197 | [-8, -62, 8] |
|  | Insula_L | 187 | [-38, -14, -2] |
|  | Rolandic_Oper_R | 184 | [58, -20, 18] |
|  | Parietal_Sup_L | 181 | [-18, -48, 76] |
|  | Postcentral_L | 163 | [-62, -18, 32] |
|  | Thalamus_R | 158 | [12, -14, 12] |
|  | Parietal_Sup_R | 153 | [22, -46, 68] |
|  | Frontal_Sup_R | 145 | [32, -6, 60] |
|  | Rolandic_Oper_L | 145 | [-42, -6, 4] |
|  | Supp_Motor_Area_R | 145 | [16, 4, 66] |
|  | Supp_Motor_Area_L | 125 | [0, 0, 48] |
|  | Thalamus_L | 121 | [-8, -18, 12] |
|  | Lingual_L | 110 | [-2, -68, 6] |
|  | Cingulum_Ant_L | 105 | [0, 8, 30] |
|  | Temporal_Sup_R | 105 | [58, -4, 4] |
|  | Paracentral_Lobule_R | 101 | [10, -42, 68] |
|  | Frontal_Mid_R | 73 | [36, 52, 24] |
|  | Lingual_R | 59 | [20, -46, -2] |
|  | Cerebelum_Crus1_L | 59 | [-22, -68, -30] |
| **funcSurg PCA comp3** | Postcentral_L | 729 | [-18, -30, 70] |
|  | Calcarine_L | 504 | [2, -70, 18] |
|  | Postcentral_R | 474 | [32, -38, 62] |
|  | Calcarine_R | 420 | [10, -80, 10] |
|  | Precentral_R | 418 | [48, -18, 60] |
|  | Cingulum_Mid_L | 379 | [0, -4, 40] |
|  | Cingulum_Mid_R | 379 | [2, -28, 50] |
|  | Supp_Motor_Area_L | 376 | [0, -16, 58] |
|  | Insula_R | 340 | [32, -24, 16] |
|  | Rolandic_Oper_R | 329 | [62, 4, 8] |
|  | Temporal_Sup_L | 329 | [-54, -28, 16] |
|  | Supp_Motor_Area_R | 304 | [2, -16, 52] |
|  | Thalamus_L | 288 | [-6, -16, 4] |
|  | Insula_L | 277 | [-36, -22, 14] |
|  | Rolandic_Oper_L | 274 | [-38, -18, 18] |
|  | Thalamus_R | 273 | [6, -24, 2] |
|  | SupraMarginal_R | 255 | [62, -30, 24] |
|  | Paracentral_Lobule_L | 238 | [-2, -30, 50] |
|  | SupraMarginal_L | 234 | [-66, -22, 18] |
|  | Precuneus_L | 233 | [-4, -46, 64] |
|  | Precentral_L | 224 | [-22, -26, 70] |
|  | Paracentral_Lobule_R | 199 | [12, -40, 58] |
|  | Precuneus_R | 192 | [2, -40, 56] |
|  | Parietal_Sup_L | 158 | [-20, -46, 62] |
|  | Frontal_Sup_R | 153 | [16, -10, 70] |
|  | Temporal_Sup_R | 149 | [56, -26, 16] |
|  | Cuneus_R | 147 | [20, -70, 20] |
|  | Cuneus_L | 129 | [2, -76, 20] |
|  | Lingual_L | 119 | [-2, -64, 8] |
|  | Frontal_Mid_L | 113 | [-28, 42, 34] |
|  | Frontal_Mid_R | 80 | [40, 46, 6] |
|  | Lingual_R | 79 | [24, -52, 2] |
|  | Cingulum_Ant_L | 72 | [-6, 22, 30] |
|  | Heschl_L | 72 | [-38, -24, 8] |
|  | Parietal_Sup_R | 68 | [16, -50, 64] |
|  | Frontal_Sup_L | 64 | [-14, -6, 72] |
|  | Frontal_Inf_Oper_R | 63 | [62, 12, 14] |
|  | Frontal_Sup_Medial_L | 50 | [-8, 16, 42] |
| **funcSurg PCA comp 4** | Postcentral_L | 851 | [-18, -30, 64] |
|  | Precentral_R | 576 | [22, -24, 64] |
|  | Postcentral_R | 475 | [18, -32, 62] |
|  | Calcarine_L | 449 | [-10, -62, 10] |
|  | Supp_Motor_Area_R | 438 | [4, 0, 46] |
|  | Cingulum_Mid_L | 405 | [-2, -2, 48] |
|  | Calcarine_R | 403 | [12, -64, 8] |
|  | Cingulum_Mid_R | 397 | [8, -30, 46] |
|  | Supp_Motor_Area_L | 396 | [-4, 0, 46] |
|  | SupraMarginal_L | 336 | [-50, -40, 28] |
|  | Insula_R | 322 | [34, -20, 18] |
|  | Precuneus_R | 320 | [8, -38, 54] |
|  | Rolandic_Oper_R | 297 | [48, -16, 12] |
|  | Thalamus_L | 291 | [-4, -24, 8] |
|  | Rolandic_Oper_L | 287 | [-38, -28, 18] |
|  | Precentral_L | 268 | [-26, -24, 68] |
|  | Thalamus_R | 263 | [14, -26, 8] |
|  | Insula_L | 261 | [-36, -18, 20] |
|  | Precuneus_L | 260 | [-12, -42, 68] |
|  | Paracentral_Lobule_L | 248 | [-16, -32, 64] |
|  | SupraMarginal_R | 245 | [44, -32, 24] |
|  | Temporal_Sup_L | 229 | [-40, -16, -6] |
|  | Paracentral_Lobule_R | 202 | [10, -42, 66] |
|  | Lingual_L | 174 | [0, -80, 4] |
|  | Parietal_Sup_L | 156 | [-26, -48, 64] |
|  | Temporal_Sup_R | 151 | [54, -32, 22] |
|  | Cuneus_L | 149 | [0, -74, 18] |
|  | Frontal_Sup_R | 137 | [22, -4, 66] |
|  | Lingual_R | 132 | [16, -50, 0] |
|  | Cuneus_R | 129 | [4, -84, 34] |
|  | Parietal_Sup_R | 124 | [22, -50, 74] |
|  | Frontal_Mid_R | 76 | [50, 20, 40] |
|  | Cingulum_Ant_L | 61 | [0, 4, 30] |
|  | Cerebelum_Crus1_L | 59 | [-22, -68, -30] |
|  | Frontal_Inf_Oper_L | 58 | [-42, 10, 6] |

**Table S3 PCA-derived rs-fMRI maps results**. Brain regions, identified with the Automated Anatomical Labelling (AAL) Atlas, emerged from the PCA-derived rs-fMRI maps. Peak coordinates are expressed in MNI space. Regions were reported when the number of significant voxels was> 50. Abbreviations: rt-fMRI-NF, real-fMRI neurofeedback; funcSurg, functional surgery; PCA, principal component analysis; comp, component. R, right; L, left.

**Table S4**

| **rt-fMRI-NF PCA comp1** | | **rt-fMRI-NF PCA comp2** | | **funcSurg PCA comp1** | | **funcSurg PCA comp2** | | **funcSurg PCA comp3** | | **funcSurg PCA comp4** | |
| --- | --- | --- | --- | --- | --- | --- | --- | --- | --- | --- | --- |
| **terms** | ***r*** | **terms** | ***r*** | **terms** | ***r*** | **terms** | ***r*** | **terms** | ***r*** | **terms** | ***r*** |
|  |  |  |  |  |  |  |  |  |  |  |  |
| cingulate cortex | 0.37 | somatosensory | 0.36 | painful | 0.26 | supramarginal g. | 0.06 | thalamic | 0.11 | primary motor | 0.09 |
| cingulate | 0.36 | stimulation | 0.30 | pain | 0.25 | supramarginal | 0.06 | thalamus | 0.09 | sensorimot. cort. | 0.09 |
| anterior cingulate | 0.34 | sensorimotor | 0.28 | sec. somatosens. | 0.21 | depression | 0.04 | cortex thalamus | 0.05 | motor cortex | 0.09 |
| cortex acc | 0.27 | motor cortex | 0.27 | somatosens. | 0.21 | empathy | 0.04 | detected | 0.05 | sensorimotor | 0.09 |
| acc | 0.27 | primary motor | 0.26 | insula | 0.19 | action observation | 0.04 | sighted | 0.04 | somatosensory | 0.08 |
| money | 0.13 | somatosens. cortex | 0.25 | somatosens. cort. | 0.18 | inferior parietal | 0.04 | visual cortex | 0.04 | cortex m1 | 0.07 |
| posterior cingulate | 0.25 | posterior insula | 0.25 | stimulation | 0.18 | precuneus | 0.04 | somatosensory | 0.03 | motor | 0.07 |
| default mode | 0.22 | sec. somatosens. | 0.25 | sii | 0.18 | actions | 0.03 | cortex m1 | 0.03 | m1 | 0.07 |
| vmpfc | 0.22 | sii | 0.25 | anterior insula | 0.17 | temporal | 0.03 | primary visual | 0.03 | thalamic | 0.07 |
| ventromedial pref | 0.21 | primary somatosens. | 0.24 | posterior insula | 0.16 | orienting | 0.03 | nociceptive | 0.02 | movement | 0.07 |
| mpfc | 0.21 | somatosens. Cort. | 0.24 | somatosens. cort | 0.16 | medial prefrontal | 0.03 | sensorimotor net. | 0.02 | motor premotor | 0.07 |
| prefrontal cortex | 0.19 | sensory | 0.24 | insular | 0.15 | empathic | 0.03 | somatosens. cort. | 0.02 | motor sma | 0.06 |
| referential | 0.19 | sensorimot. Cort. | 0.24 | sensorimotor | 0.11 | languages | 0.02 | reho | 0.02 | supp. motor | 0.06 |
| cortex vmpfc | 0.18 | speech production | 0.23 | operculum | 0.14 | efficiency | 0.02 | values | 0.02 | prim. Sensorimot. | 0.06 |
| pcc | 0.18 | motor | 0.23 | motor | 0.14 | semantic | 0.02 | lingual | 0.02 | rehabilitation | 0.06 |
| cortex mpfc | 0.18 | s1 | 0.22 | noxious | 0.14 | pfc | 0.02 | smoking | 0.02 | thalamus | 0.06 |
| dmn | 0.17 | production | 0.21 | supp. motor | 0.13 | objects | 0.02 | motor function | 0.06 | motor function | 0.06 |
| network dmn | 0.17 | si | 0.21 | sensorimotor | 0.11 | intraparietal sulcus | 0.02 | movements | 0.05 | movements | 0.05 |
| self referential | 0.17 | auditory cortex | 0.20 | s1 | 0.13 | sec. somatosens. | 0.02 | motor task | 0.05 | motor task | 0.05 |
| reward | 0.14 | movement | 0.19 | insula anterior | 0.13 | lingual gyrus | 0.02 | stimulation tms | 0.05 | stimulation tms | 0.05 |
| value | 0.13 | primary auditory | 0.19 | insular cortex | 0.12 | sleep | 0.02 | somatosens. cort. | 0.02 | stroke | 0.05 |

**Table S4. Neurosynth decoding of the PCA rs-fMRI-derived maps.** Only the first top 20 terms were retained. Abbreviations: rt-fMRI-NF, real-fMRI neurofeedback; funcSurg, functional surgery; PCA, principal component analysis; comp, component; cort., cortex or cortices; somatosens., somatosensory; sensorimot., sensorimotor; sec., secondary.

**Table S5**

| Term | AAL Region | # Voxels | Peak coordinate [MNI] |
| --- | --- | --- | --- |
| **Pain** | Insula_L | 1467 | [-34, 18, 4] |
|  | Insula_R | 1450 | [38, 22, 0] |
|  | SupraMarginal_R | 932 | [54, -28, 24] |
|  | Rolandic_Oper_R | 870 | [54, -26, 22] |
|  | Cingulum_Mid_R | 709 | [2, 20, 32] |
|  | Rolandic_Oper_L | 708 | [-38, -18, 16] |
|  | Thalamus_L | 697 | [-8, -14, 4] |
|  | Cingulum_Mid_L | 668 | [2, 18, 34] |
|  | Thalamus_R | 656 | [10, -14, 6] |
|  | Postcentral_L | 639 | [-60, -22, 22] |
|  | SupraMarginal_L | 634 | [-58, -22, 22] |
|  | Supp_Motor_Area_R | 608 | [4, 14, 50] |
|  | Supp_Motor_Area_L | 592 | [-2, 8, 44] |
|  | Frontal_Inf_Oper_R | 578 | [42, 12, 4] |
|  | Cingulum_Ant_L | 539 | [-2, 22, 28] |
|  | Putamen_R | 495 | [32, 10, 8] |
|  | Frontal_Mid_R | 487 | [46, 42, 4] |
|  | Frontal_Inf_Tri_R | 422 | [42, 18, 4] |
|  | Temporal_Sup_L | 386 | [-56, -26, 16] |
|  | Cingulum_Ant_R | 363 | [2, 20, 28] |
|  | Putamen_L | 344 | [-14, 10, 0] |
|  | Parietal_Inf_R | 324 | [42, -44, 44] |
|  | Temporal_Sup_R | 309 | [58, -24, 16] |
|  | Parietal_Inf_L | 295 | [-54, -28, 36] |
|  | Postcentral_R | 290 | [62, -16, 20] |
|  | Frontal_Inf_Oper_L | 284 | [-38, 8, 10] |
|  | Frontal_Sup_Medial_L | 282 | [2, 18, 42] |
|  | Precentral_L | 268 | [-34, -28, 56] |
|  | Frontal_Inf_Tri_L | 245 | [-36, 20, 8] |
|  | Amygdala_L | 186 | [-22, -6, -16] |
|  | Frontal_Inf_Orb_R | 162 | [36, 22, -8] |
|  | Amygdala_R | 159 | [24, -2, -18] |
|  | Hippocampus_L | 155 | [-22, -10, -16] |
|  | Frontal_Inf_Orb_L | 145 | [-38, 22, -4] |
|  | Hippocampus_R | 136 | [22, -2, -20] |
|  | Precentral_R | 131 | [48, 6, 36] |
|  | Frontal_Sup_Medial_R | 131 | [6, 22, 44] |
|  | Pallidum_L | 127 | [-14, 8, 0] |
|  | Caudate_R | 117 | [12, 10, 2] |
|  | Frontal_Med_Orb_L | 112 | [-2, 54, -10] |
|  | Pallidum_R | 108 | [18, 6, -2] |
|  | Heschl_R | 108 | [42, -18, 14] |
|  | Frontal_Med_Orb_R | 96 | [2, 38, -14] |
|  | Temporal_Pole_Sup_L | 92 | [-50, 6, 0] |
|  | Heschl_L | 91 | [-38, -18, 10] |
|  | Cerebelum_6_L | 86 | [-26, -66, -24] |
|  | Temporal_Mid_L | 68 | [-48, -68, 0] |
|  | Temporal_Pole_Sup_R | 67 | [58, 8, 0] |
|  | Frontal_Mid_L | 62 | [-32, 52, 20] |
|  | Caudate_L | 62 | [-10, 10, 0] |
|  | Temporal_Mid_R | 61 | [52, -66, 0] |
|  | Occipital_Mid_L | 54 | [-48, -68, 4] |
| **Chronic pain** | Insula_R | 600 | [36, 6, 6] |
|  | Insula_L | 475 | [-34, 22, 0] |
|  | Thalamus_L | 389 | [-10, -18, 6] |
|  | Thalamus_R | 286 | [10, -16, 2] |
|  | SupraMarginal_R | 188 | [58, -22, 22] |
|  | Putamen_R | 181 | [22, 10, -6] |
|  | Putamen_L | 146 | [-28, 2, -2] |
|  | Cingulum_Mid_L | 142 | [2, 12, 40] |
|  | Rolandic_Oper_L | 118 | [-40, -20, 14] |
|  | Supp_Motor_Area_L | 111 | [-2, 8, 44] |
|  | Rolandic_Oper_R | 103 | [56, -22, 22] |
|  | Caudate_L | 102 | [-10, 10, 0] |
|  | SupraMarginal_L | 99 | [-56, -26, 24] |
|  | Amygdala_L | 97 | [-22, -2, -18] |
|  | Cingulum_Mid_R | 94 | [2, 4, 40] |
|  | Amygdala_R | 80 | [24, -2, -18] |
|  | Caudate_R | 74 | [18, 14, 2] |
|  | Frontal_Inf_Oper_R | 73 | [60, 10, 8] |
|  | Cingulum_Ant_L | 69 | [-2, 22, 28] |
|  | Parietal_Inf_R | 64 | [54, -38, 48] |
|  | Parietal_Inf_L | 61 | [-44, -44, 50] |
|  | Frontal_Inf_Tri_L | 58 | [-38, 24, 0] |
|  | Hippocampus_R | 52 | [22, -4, -20] |
|  | Postcentral_R | 52 | [56, -22, 30] |

**Table S5. Neurosynth Uniformity maps results for the terms ''pain' and 'chronic pain'.** Brain regions, identified with the Automated Anatomical Labelling (AAL) Atlas, emerged from Uniformity maps. Peak coordinates are expressed in MNI space. Regions were reported when the number of significant voxels was> 50. Abbreviations: rt-fMRI-NF, real-fMRI neurofeedback; funcSurg, functional surgery; PCA, principal component analysis; comp, component. R, right; L, left.

| **Table S6** | | | | | | |
| --- | --- | --- | --- | --- | --- | --- |
|  | **funcSurg PCA comp1** | **funcSurg PCA comp2** | **funcSurg PCA comp3** | **funcSurg PCA comp4** | **rt-fMRI-NF PCA comp1** | **rt-fMRI-NF PCA comp1** |
| **PET Map** | *p-value* | *p-value* | *p-value* | *p-value* | *p-value* | *p-value* |
| 5HT1a(WAY) | 0.018 | 0.571 | 0.760 | 0.905 | 0.686 | 0.433 |
| 5HT1a(cumi) | 0.140 | 0.459 | 0.732 | 0.857 | 0.713 | 0.884 |
| 5HT1b(P943) | 0.001** | 0.898 | 0.061 | 0.065 | 0.111 | 0.379 |
| 5HT1b(az) | 0.060 | 0.611 | 0.844 | 0.356 | 0.183 | 0.456 |
| 5HT2a(ALT) | 0.001** | 0.683 | 0.169 | 0.094 | 0.060 | 0.739 |
| 5HT2a(cimbi) | 0.010 | 0.464 | 0.599 | 0.173 | 0.116 | 0.795 |
| 5HT4(sb20) | 0.485 | 0.893 | 0.835 | 0.931 | 0.627 | 0.819 |
| CB1(PFMPEPd2) | 0.001** | 0.308 | 0.853 | 0.416 | 0.023 | 0.849 |
| D1(SCH23390) | 0.326 | 0.556 | 0.201 | 0.457 | 0.057 | 0.516 |
| D2(RACLOPRIDE) | 0.141 | 0.350 | 0.219 | 0.060 | 0.321 | 0.232 |
| D2(fallypride) | 0.615 | 0.562 | 0.831 | 0.721 | 0.567 | 0.212 |
| DAT(DATSPECT) | 0.092 | 0.114 | 0.953 | 0.918 | 0.007 | 0.530 |
| FDOPA(f18) | 0.090 | 0.128 | 0.927 | 0.823 | 0.001** | 0.473 |
| GABAa(FLUMAZENIL) | 0.001** | 0.386 | 0.112 | 0.015 | 0.141 | 0.475 |
| GABAa(flumazenil) | 0.051 | 0.974 | 0.776 | 0.205 | 0.234 | 0.656 |
| KappaOp(LY2795050) | 0.001** | 0.518 | 0.562 | 0.206 | 0.018 | 0.205 |
| MU(CARFENTANIL) | 0.952 | 0.464 | 0.136 | 0.763 | 0.471 | 0.950 |
| MU(carfentanil) | 0.452 | 0.659 | 0.280 | 0.835 | 0.864 | 0.862 |
| NAT(MRB) | 0.001** | 0.001** | 0.323 | 0.001** | 0.019 | 0.001** |
| NMDA(ge179) | 0.740 | 0.041 | 0.156 | 0.046 | 0.551 | 0.211 |
| SERT(DASB) | 0.572 | 0.043 | 0.155 | 0.775 | 0.002 | 0.946 |
| SERT(MADAM) | 0.823 | 0.130 | 0.187 | 0.529 | 0.034 | 0.744 |
| SERT(dasb) | 0.269 | 0.082 | 0.611 | 0.691 | 0.015 | 0.858 |
| VAChT(feobv1) | 0.586 | 0.028 | 0.243 | 0.526 | 0.437 | 0.003 |
| VAChT(feobv2) | 0.111 | 0.012 | 0.582 | 0.899 | 0.005 | 0.027 |
| VAChT(feobv3) | 0.001** | 0.086 | 0.193 | 0.456 | 0.027 | 0.056 |
| mGluR5(abp1) | 0.003 | 0.638 | 0.443 | 0.267 | 0.348 | 0.471 |
| mGluR5(abp2) | 0.001** | 0.963 | 0.284 | 0.185 | 0.525 | 0.353 |
| mGluR5(abp3) | 0.001** | 0.968 | 0.392 | 0.115 | 0.271 | 0.378 |
| **Table S6 Neurotransmitter receptors profiling of Principal component analysis (PCA) rs-fMRI-derived maps**. P-values of the spatial correlation analyses between each PCA rs-fMRI-derived map and the neurotransmitter receptor distribution maps obtained with Juspace (Dukart et al., 2020). Abbreviations: funcSurg, functional neurosurgery; rt-fMRI-NF, real-time fMRI neurofeedback; PCA, principal component analysis; comp, component. ***p*-value < 0.0017, Bonferroni corrected for 29 statistical tests. For an extended explanation of the neurotransmitter maps employed, see Dukart et al. (2020). | | | | | | |

**Captions**

**Figure S1.** Flowchart of the screening process of the rt-fMRI-NF studies according to the PRISMA guideline.


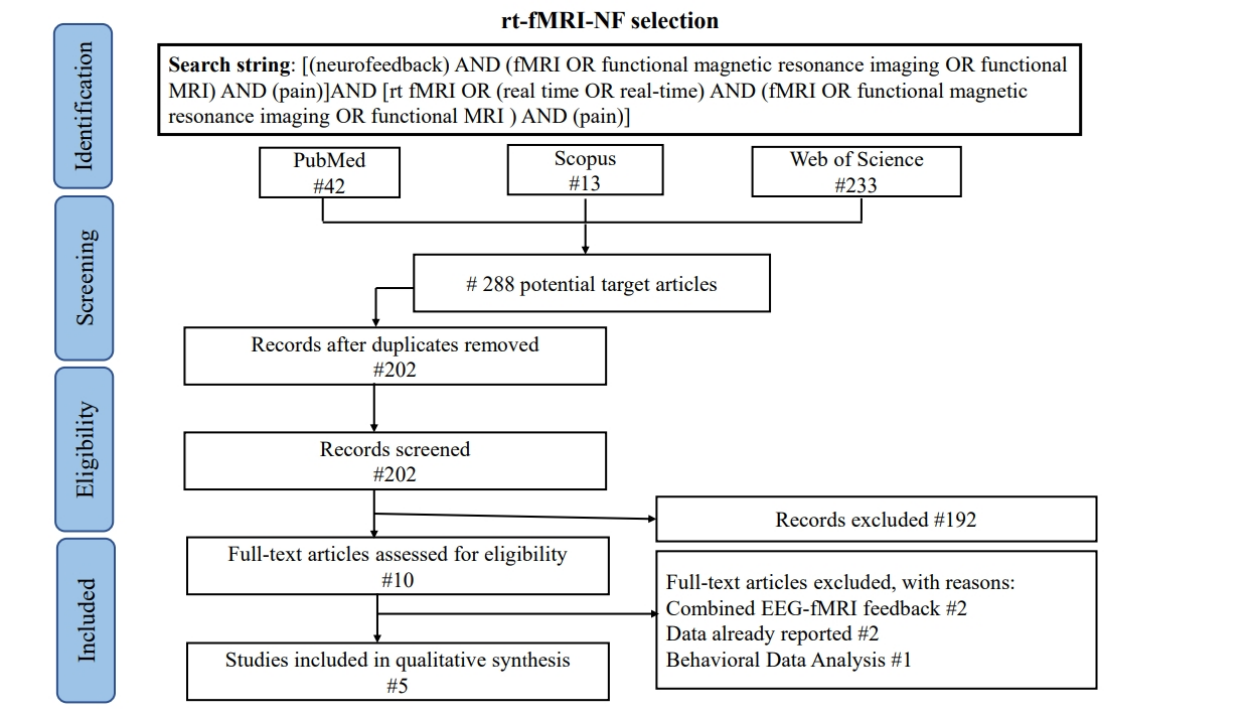


**Figure S2.** Flowchart of the screening process of the meta-analyses according to the PRISMA guideline.


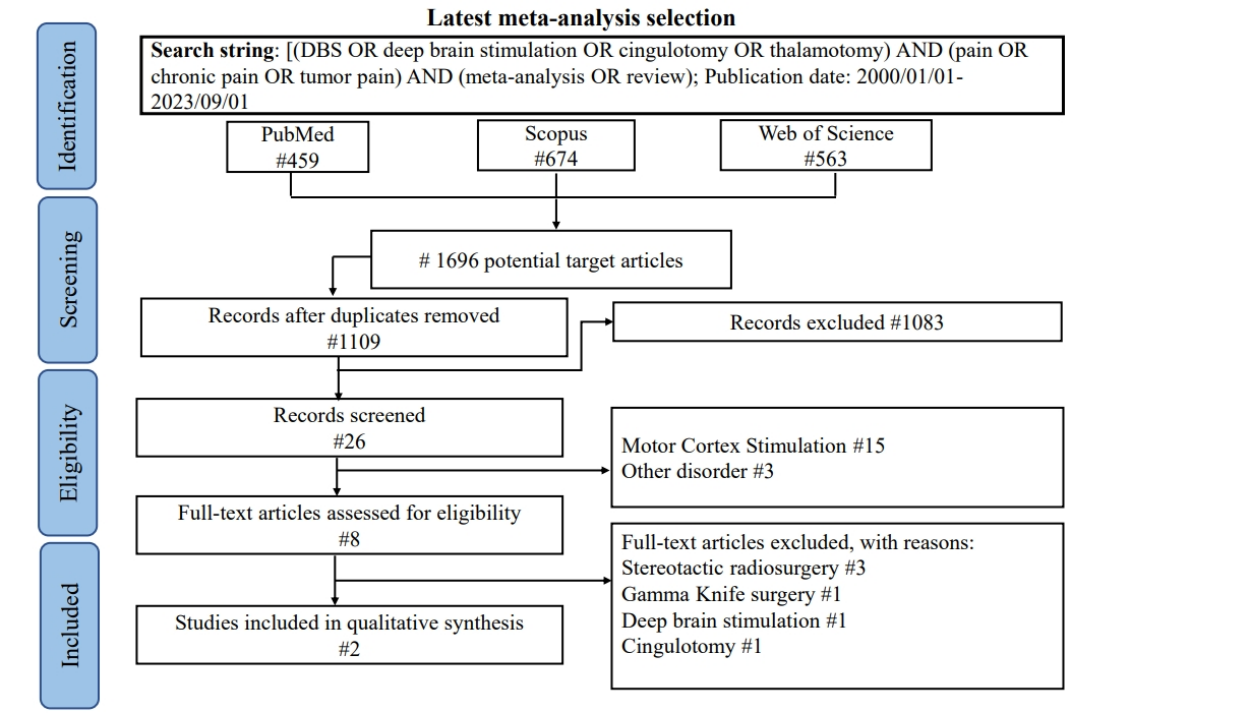


**Figure S3.** Flowchart of the screening process of the DBS studies according to the PRISMA guideline
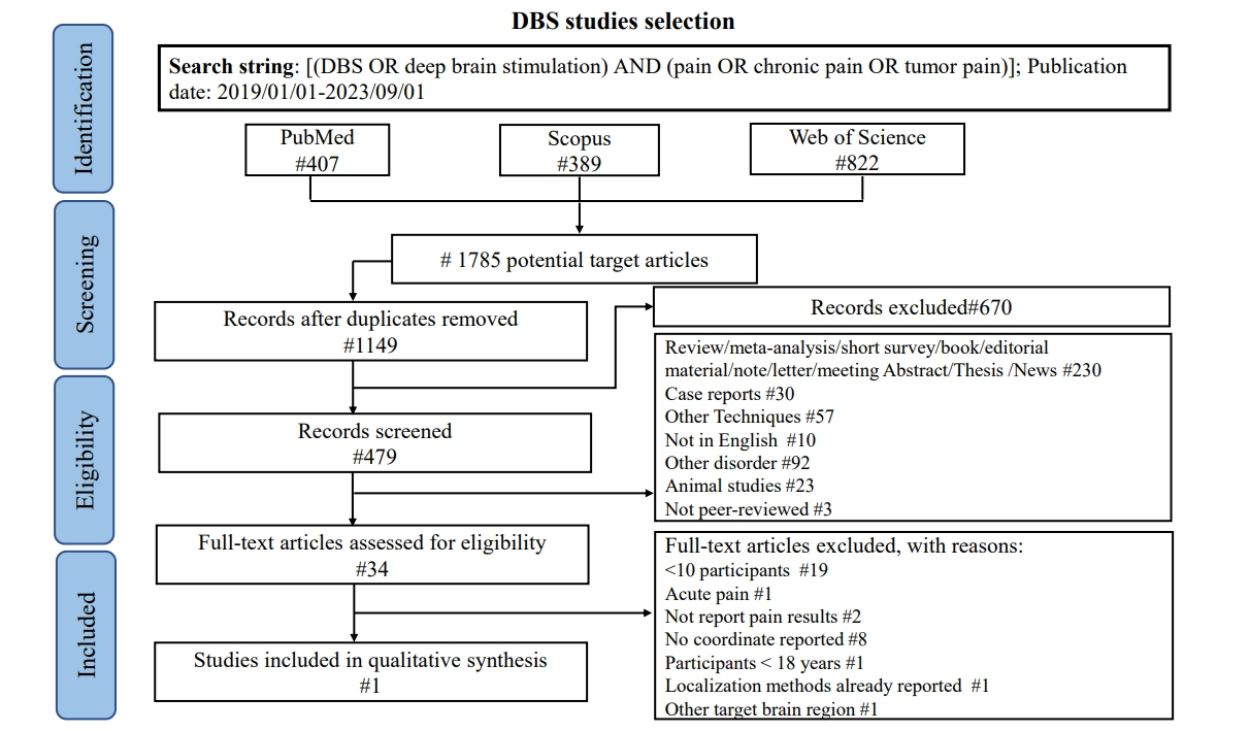


**Figure S4.** Flowchart of the screening process of the Cingulotomy studies according to the PRISMA guideline
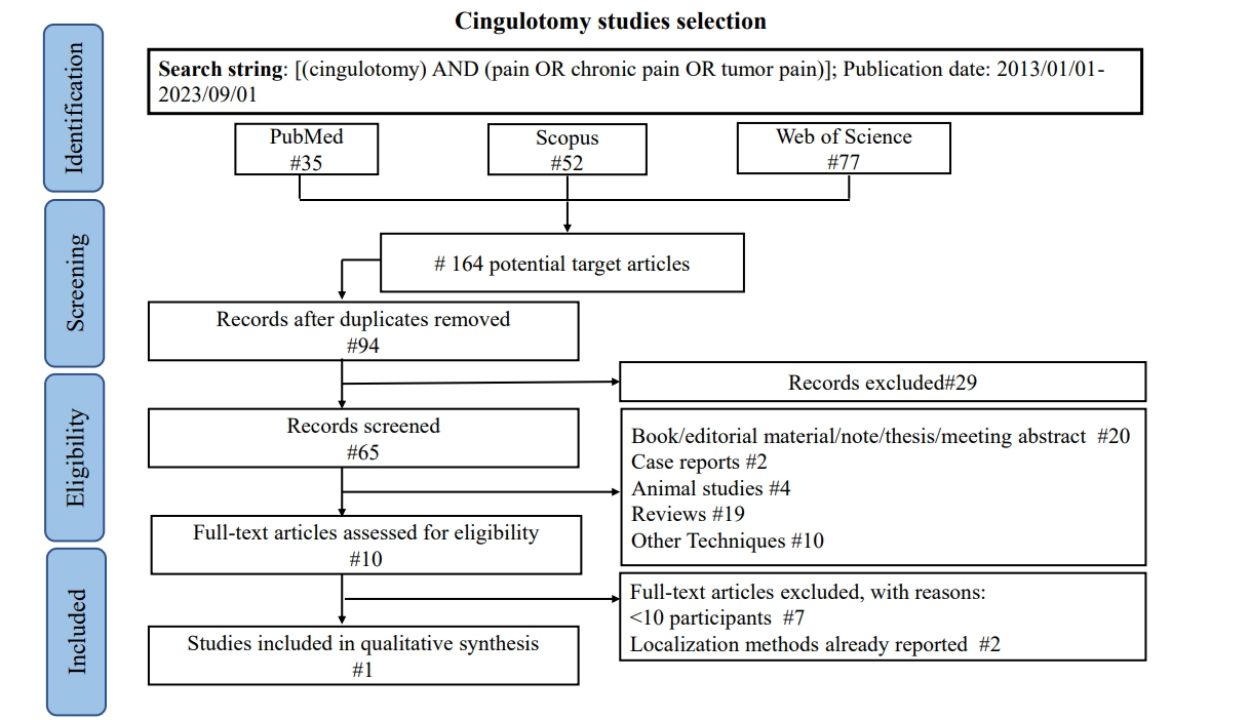


**Figure S5.** Flowchart of the screening process of the Thalamotomy studies according to the PRISMA guideline.


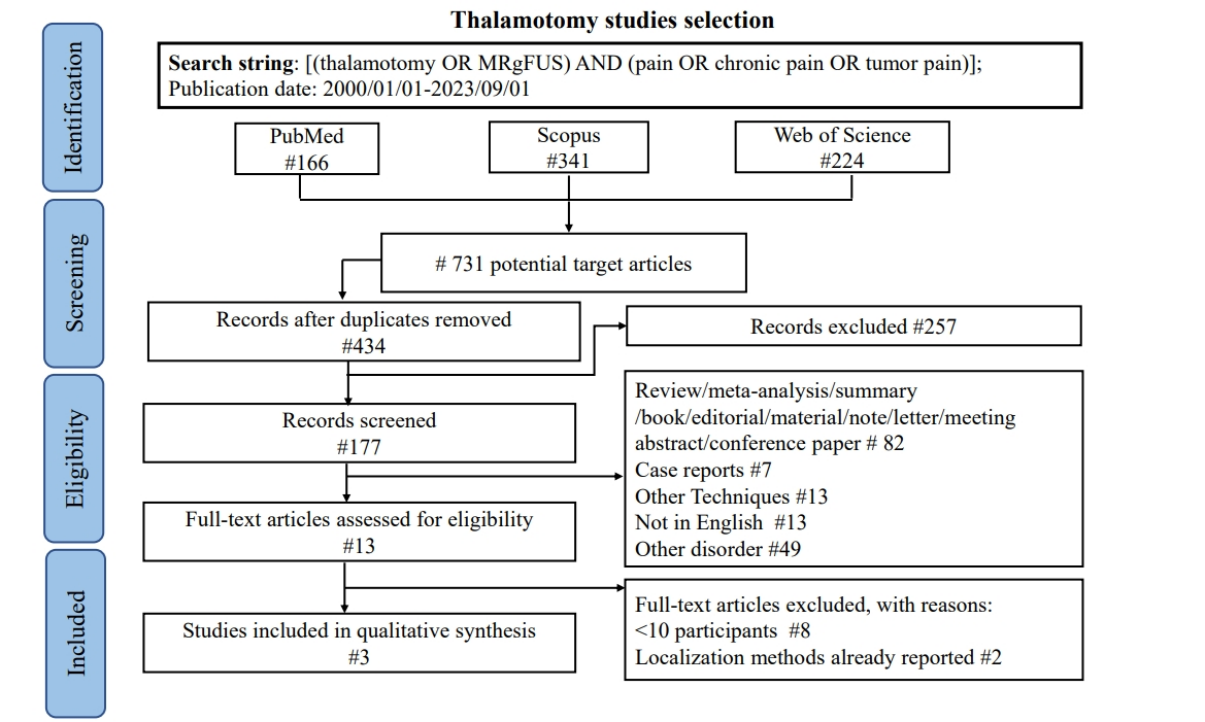


**Figure S6.** Correlation coefficients between neurotransmitter receptor distribution maps available in Juspace and PCA rs-fMRI-derived maps. For an extended explanation of the neurotransmitter maps employed, see Dukart et al. (2020).


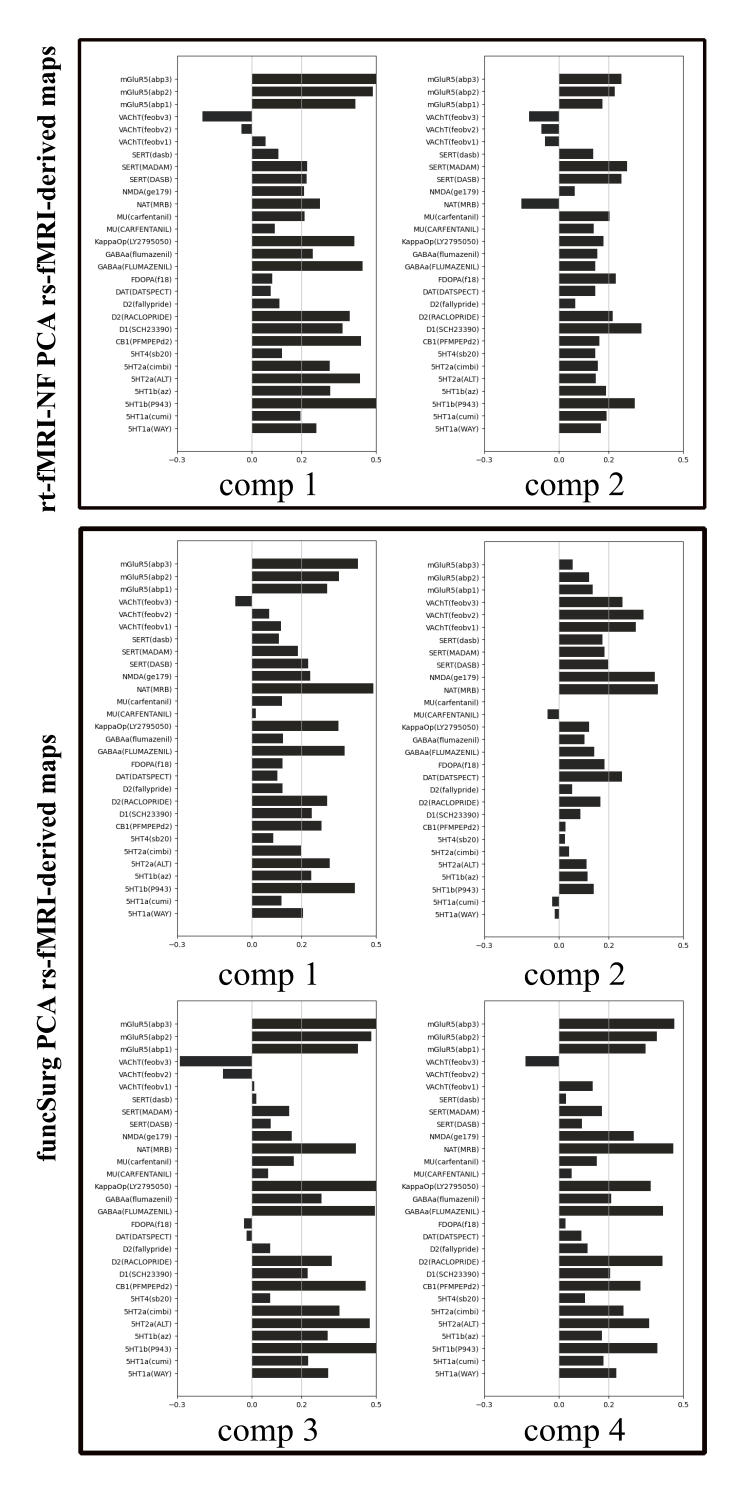

Supplement: kkae026_Supplemental_File [file kkae026_supplemental_file.docx]
